# Stepwise phage resistance and collateral phage susceptibility in *Klebsiella pneumoniae*

**DOI:** 10.1080/22221751.2026.2648890

**Published:** 2026-03-19

**Authors:** Xin Yin, Yu Feng, Huan Luo, Qingqing Fang, Jing Yu, Alan McNally, Zhiyong Zong

**Affiliations:** a Center of Infectious Diseases, West China Hospital, Sichuan University, Chengdu, People’s Republic of China; b Laboratory of Pathogen Research, West China Hospital, Sichuan University, Chengdu, People’s Republic of China; c Division of Infectious Diseases, State Key Laboratory of Biotherapy, Chengdu, People’s Republic of China; d Experimental Public Platform Management Section, West China Hospital, Sichuan University, Chengdu, People’s Republic of China; e Medical Center of General Practice, West China Hospital, Sichuan University, Chengdu, People’s Republic of China; f Animal Disease Prevention and Food Safety Key Laboratory of Sichuan Province, Key Laboratory of Bio-Resource and Eco-Environment of Ministry of Education, College of Life Sciences, Sichuan University, Chengdu, People’s Republic of China; g Institute of Microbiology and Infection, College of Medicine and Health, University of Birmingham, Birmingham, UK; h State Key Laboratory of Respiratory Health and Multimorbidity, Chengdu, People’s Republic of China

**Keywords:** Antimicrobial resistance, *Klebsiella pneumoniae*, carbapenem resistance, phage therapy, phage resistance, capsule, insertion sequence

## Abstract

Carbapenem-resistant *Klebsiella pneumoniae* (CRKP) is a difficult-to-treat pathogen. Phages recovered by a “stepwise” approach may constitute a cocktail able to prolong retardation of bacterial regrowth. We stepwise recovered three lytic phages (namely P04, P40, and P49) from different families against ST11KL64 CRKP and created a cocktail that restrained CRKP growth for 15 h. Phage P04 recognized bacterial capsular polysaccharide (CPS). P04/P40-resistant mutants lost the large fragments containing CPS gene due to homologous recombination between two insertion sequences. Through gene cloning and complementation experiments, CPS and lipopolysaccharide (LPS) quantification, and untargeted lipid metabolism assays, deletion of the *ugd*/*wbgU* genes alters lipid A modification and outer membrane environment. This change affects the accessibility of an unidentified membrane protein or lipid A itself, which serves as the receptor for P40. P49 recognized bacterial transmembrane protein involved in vitamin B_12_ transportation. Synergistic antibacterial activity was observed because the three phages recognize different receptors. Notably, P49 also lysed *Salmonella enterica*, *Escherichia coli*, *Enterobacter ludwigii*, and *Kluyvera tianfuensis*, suggesting new receptors would be exposed when the synthesis of CPS was inhibited, thereby allowing efficient attack by phages originally targeting other species (the “close-one-door-but-open-another” phenomenon). The conserved structure of BtuB provides a molecular basis for the rare cross-genus activity of P49. Insertion sequences provide a generalized anti-phage defence in encapsulated bacteria. Our findings provide critical insights into the versatile mechanisms underpinning bacteria-phages interactions. Specifically, the collateral susceptibility to phages targeting other bacterial species may provide a novel and highly promising approach for creating clinically viable phage cocktails.

## GRAPHICAL ABSTRACT

**Figure:**
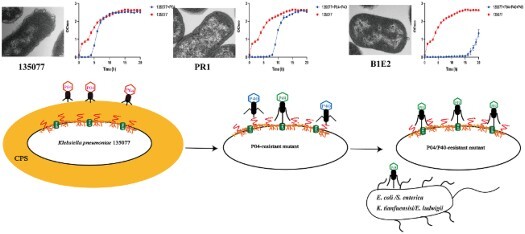

## Introduction

With the use and abuse of antimicrobial agents, carbapenem-resistant *Klebsiella pneumoniae* (CRKP) has emerged and spread globally, listed by WHO as one of the critical priority antibiotic-resistant pathogens [1]. The annual number of deaths attributable to CRKP increased by more than 25,000 from 1990 to 2021, which results in an enormous health and economic burden [2]. The population structure of CRKP varies geographically. Sequence type (ST) 258 is predominant in North America, Latin America, and Europe [3,4]. ST11 is the most prevalent CRKP type in China [5], and can be further assigned to several capsule types, among which KL64 is the major capsule type of ST11 CRKP [6].

Therapeutic options for CRKP infections are limited, urging the development of novel treatment regimens to treat this infection successfully. An increasing number of physicians and scientists advocate for phage therapy to help overcome the antimicrobial resistance crisis [7]. At present, the United States, Belgium, France, and Sweden have established phage therapy programmes [8]. Phage products targeting multiple bacterial pathogens have been used in the clinic [9,10]. However, whether synergy occurs among clinically used phages remains unclear and the emergence of phage-resistant mutants is a yet-to-be-overcome bottleneck compromising the efficacy of phage therapy [11]. It has been reported that repeated use of the same phage reduced the therapeutic efficacy of phages [12,13]. Therefore, it has been proposed to use phage cocktails, with clarified synergistic effects, as a potential approach to combat multidrug-resistant bacterial infection. Additionally, it may be beneficial to minimize premature exposure to certain phages, similar to the precautions taken against the misuse of antibiotics [12,13].

Although phages provide a potential antibacterial treatment, *Klebsiella pneumoniae* (KP) is also evolving adaptive mechanism to resist phage predation. KP defends against phage infection through various strategies, including receptor modification, restriction-modification systems [14], toxin-antitoxin systems [15], and CRISPR-Cas systems [16]. Among these, receptor-blocking mechanism is the primary cause of phage resistance in KP, and capsular polysaccharide (CPS) of KP is the major receptor for phage adsorption [17]. After the loss of CPS, KP exposes more receptors such as lipopolysaccharide (LPS) [18] and outer membrane proteins [19]. Phage cocktail targeting different receptors can reduce the emergence of phage-resistant mutants [18]. Furthermore, by targeting more conserved receptors, the phage can develop cross-genus infection and expand the host range.

The host specificity of phages is widely recognized as a fundamental property that significantly influences their therapeutic potential [20]. The CPS, the outermost structure of KP [21], serves as a crucial determinant of the phage host range [22]. Therefore, most phages target and lyse KP by recognizing CPS, while even changes in acetylation levels of CPS can decrease phage adsorption [23]. Therapeutic phages with the ability to lyse bacterial strains across species are still uncommon in the literature [24,25]. Interestingly, we found that a phage recovered using a CPS- and LPS-deficient KP strain could infect cross-genus bacteria. We speculate that the loss of CPS in KP may expose more conserved receptors, allowing phages to expand the host range. Broad-spectrum phages, due to their ability to target a broader range of bacteria, have greater therapeutic potential in clinical applications.

Therefore, we constructed a personalized phage cocktail and elucidated the synergistic effect of the phage cocktail by studying the receptors recognized by phage. Our findings reveal bacterial defence mechanism of bacteria against phages and offer insights that may inform engineering or modifying phages to generate more candidates with therapeutic potential. Moreover, we found that the third round of phage can also lyse *Salmonella enterica* and *Escherichia coli* in addition to phage-resistant CRKP mutants. The collateral susceptibility to phages attacking other bacterial species opens a new window for creating cocktails to overcome phage resistance.

## Materials and methods

### Strains and culturing conditions

A clinical ST11-KL64 CRKP strain, namely 135077, obtained from a urine sample in 2020 [26], was used as the host strain for isolating phages. Strain 135077 harboured no known carbapenemase genes but was resistant to meropenem (MIC, 4 µg/ml), likely due to its OmpK36 porin deficiency and production of CTX-M-65 extended-spectrum β-lactamase in combination [26]. In addition, the strain contains six intact prophage regions (Supplementary Table S1) as revealed by PHASTER (Phage Search Tool Enhanced Release) [27]. CRKP strains were grown using chromogenic agar plates (CHROMagar Orientation; CHROMagar; Paris, France) containing 2 µg/ml meropenem and 64 µg/ml linezolid (to prevent contamination of Gram-positive bacteria). Strains of other species belonging to the family *Enterobacteriaceae*, specifically, *E. coli*, *Enterobacter* spp., *Kluyvera* spp., and *S. enterica* were cultured using Luria–Bertani (LB; Sangon, Shanghai, China) agar plates. For all strains, single colonies were picked and cultured in LB broth (Sangon) at 37°C with shaking at 180 rpm. Further information is available in the supplementary materials.

### Isolation of phage and phage-resistant mutants

Phage isolation was conducted using the double-layer agar method as described previously [26]. Three sequential rounds of phage isolation were conducted. In the first round, 135077 was used as a host bacterial strain and phage 150004 (assigned P04 here for brevity) was isolated [26]. In the second round, a P04-resistant mutant, PR1 [26], was used as the host strain to isolate lytic phages. River water was collected from the Jinjiang River (Chengdu, China) in July 2021, from which phage 150040 (assigned P40 here for brevity) was isolated. To isolate new phages that could prolong the antibacterial activity, we cocultured strain 135077 with phages P04 and P40 and therefore obtained three P04/P40-resistant mutants named B1E1, B1E2, and B1E3, respectively. In the third round, a P04/P40-resistant mutant, B1E2, was used as the host strain to isolate lytic phages. P49 was recovered from the untreated influx of the wastewater processing station at West China Hospital collected in January 2022. We also cocultured P04, P40, and P49 with strain 135077 and obtained two P04/P40/P49-resistant mutants named B1G1 and B1G2. We also isolated phage-resistant mutants against two distinct ST11-KL64 CRKP clinical isolates (strains 140494 and 140443). The detailed procedure is described in the supplementary materials.

### Biological characterization of phages

We performed biological characterization of phages as previously reported [26], including the optimal multiplicity of infection, adsorption rate, one-step growth curve, pH, and thermal stability, and transmission electron microscopy. The method detail is shown in the supplementary materials.

### Phage host range determination

We used an additional 31 CRKP strains (Supplementary Table S2), including 12 STs and 17 capsular (KL) types, for determining the phage host range of phages P04, P40, and P49 using spot assays [28]. For examining whether the phages could lyse species other than *Klebsiella*, we also included 20 strains of the family *Enterobacteriaceae*, specifically 8 *Enterobacter* strains (four *Enterobacter hoffmannii* and one of each of *Enterobacter ludwigii, Enterobacter sichuanensis, Enterobacter xiangfangensis*), 7 *S. enterica* strains of six different serotypes (2 Enteritidis, 2 Typhimurium, and one for each of Derby, Indiana, and Thompson), three *E. coli*, and two *Kluyvera* strains (one *Kluyvera cryocrescens* and one *Kluyvera tianfuensis*) (Table S3) for the assay. Notably, *K. tianfuensis* is a new species with deposition in the SeqCode registry (https://registry.seqco.de/names/49651)*.* The presence of a clear zone and lysis plaque was recorded as the strain is susceptible to the tested phage.

### *In vitro* phage bacteriolytic assay

The bacteriolytic activity of P04 alone, P04 and P40 in combination, and three phages (P04, P40, and P49) in combination against strain 135077 was tested. The bacteriolytic activity of P49 against *E. coli* strain ATCC25922, *E. ludwigii* strain 170224, *K*. *tianfuensis* strain 142486, and *S. enterica* strains 38_AN were also assayed. Phage suspensions were added to a culture of the host strain at the logarithmic growth phase (McFarland = 0.5, about 10^8^ CFU/ml) at 1 MOI. The phage cocktail was prepared by combining two or three phages at equal ratios (1:1 or 1:1:1). The 96-well plate with the mixture was placed in an automatic microbial growth curve instrument (Scientz; Ningbo, China) for determination of their OD values at a 1-h interval for up to 20 h. Host bacterial strains cultures without phages and LB broth without bacteria were used as the positive and negative controls, respectively. The assays were performed in triplicate.

### Quantification of CPS and endotoxin

To measure the CPS production in phage-resistant mutants, we performed CPS extraction and quantification assay as described previously [26,29]. Briefly, 500 µl of bacteria culture (normalized to an OD_600_ of 2.0) was mixed with 100 µl 1% zwittergent 3–14 detergent (Macklin Biochemical; Shanghai, China) in 100 mM citric acid (pH 2.0), then incubated at 50°C for 30 min. After centrifuging at 10,000 × g for 5 min, 250 µl of the supernatant was transferred to a new EP tube. Then, 1 ml of absolute ethanol was added to EP tube and placed at 4°C overnight. The supernatant was discarded, and double-distilled water was added to dissolve CPS. The pellets were then dissolved in 200 μl of water and mixed with 1200 μl of 12.5 mM borax (Macklin) concentrated in H_2_SO_4_. Mixtures were incubated at 100°C for 5 min and cooled to room temperature. After 20 μl of 0.15% 3-hydroxydiphenol in 0.5% NaOH solution was added, the absorbance was measured at 490 nm using a spectrophotometer (Youke). LPS was prepared using the LPS Extraction Kit (iNtRON Biotechnology; Sungnam, Korea). In brief, bacterial strains were cultured to the logarithmic growth phase and collected by centrifugation. Lysis buffer was added, and the tube was shaked vigorously. Chloroform was then added and incubated at room temperature for 5 min. After another centrifugation, the supernatants were collected. Purification buffer was added, and the mixture was incubated at −20°C for 10 min. After centrifugation, the precipitate was washed with 70% ethanol. LPS was collected by centrifugation and dried. Tris-HCl buffer solution was used to dissolve the samples. Proteinase K solution (50 μl) was added, and the samples were incubated for 30 min. The endotoxin was quantified using an endotoxin quantitative detection kit (Jinshanchuan, Beijing, China) with an automatic bacterial dynamic test tube detector (Jinshanchuan) following the manufacturer’s manual.

### TEM to observe morphology of bacteria

We used TEM to observe bacterial morphology and measure the thickness of CPS. The experimental procedures were performed as described previously [30,31], with minor modifications. Briefly, the bacterial culture at the logarithmic growth phase was centrifuged at 2000 rpm for 10 min. The pellets were resuspended in phosphate-buffered saline (PBS) and transferred to a 1.5 ml EP tube. The suspension was centrifuged again, and the supernatant was removed. Then, 1 ml of fixative solution (containing 0.15% ruthenium red, 4% paraformaldehyde, and 2.5% glutaraldehyde) was added. After 5 min of fixation, the sample was centrifuged at 13,000 rpm for 10 min with subsequent removal of the supernatant. Then, 1 ml fixative solution was gently added along the tube wall for fixation at room temperature for 2 h. After this, the sample was gently washed three times with PBS and was subsequently subjected to sectioning and TEM observation in the Lilai Biomedicine Experiment Center (Chengdu, China). The thickness of CPS was measured 10 times per sample and expressed in both transverse and longitudinal sections.

### LPS analysis by sodium dodecyl-sulfate polyacrylamide gel electrophoresis (SDS-PAGE) and silver staining

SDS-PAGE gels (4–12%) were prepared using the SDS-PAGE reagent kit (Sangon). The concentration of LPS loaded onto the gels lies between 0.1 and 0.2 µg/µl. Samples (8 μl) were mixed with 2 μl of 5 × protein loading buffer (Sangon) and then boiled for 15 min. Then the sample (10 μl) and unstained protein marker (10 μl, 14.4–116 kDa, Sangon) were added to each well. Electrophoresis was performed initially at 80 V for separation, followed by 120 V until dye front migration was complete. LPS was stained using a silver staining kit (Sangon) according to manufacturer’s instructions.

### Untargeted lipid metabolism assays

The membrane lipids of P04/P49-resistant mutant B1E2 and the corresponding strain complemented *ugd* and *wbgU* together were assayed using untargeted lipid metabolism assays. Six biological replicates were used per strain. We outsourced to Shi Biomedicine Technology Co., Ltd (Wuhan, China) for this assay. In brief, the liquid sample (200 μl) and methyl tert-butyl ether (800 μl) were mixed by vortex, then mixed with prechilled methanol (240 μl) by vortex, followed by sonication for 20 min. The organic phase was collected after centrifugation and blow-dried with nitrogen. Liquid chromatography and mass spectrometry analyses were performed using an ultra-high performance liquid chromatography system (Shimadzu; Kyoto, Japan) coupled with the Q-Exactive Plus spectrometer (Thermo Scientific; Waltham, MA, USA) [32]. Lipid analyses were conducted using LipidSearch software version 4.2 (Thermo Scientific) [33]. The differential lipids were analysed by hierarchical cluster analysis and visualized in the form of heat map using R version 3.6.3.

### Whole genome sequencing and analysis

Genomic DNA of phages and phage-resistant bacterial mutants was extracted using the Phage DNA Isolation Kit (Norgen Biotek; Thorold, Canada) and Bacterial Genomic DNA Extraction Kits (Tiangen; Beijing, China), respectively, following the manufacturer’s protocols. The prepared DNA was ultrasonically sheared into 350 bp before the construction of 150-bp paired-end libraries, which were sequenced using HiSeq X10 (Illumina; San Diego, CA, USA). The generated raw reads were quality-trimmed using Trimmomatic v0.38 [34], and assembled into draft genomes using SPAdes v3.13.0 [35] under “isolate” mode.

Genome sequences were annotated using Prokka v1.12 54 [36] and Rapid Annotations Subsystems Technology (RAST, http://rast.nmpdr.org/) [37]. Unannotated protein sequences were manually analysed using the Basic Local Alignment Search Tool (BLASTp, https://blast.ncbi.nlm.nih.gov/Blast.cgi). The Virulence Factor Database (VFDB, http://www.mgc.ac.cn/VFs/main.htm) [38] and Comprehensive Antibiotic Resistance Database (CARD, https://card.mcmaster.ca/analyze/rgi) [39] were used to detect virulence factors and antimicrobial resistance genes. Phage genome maps were generated using Proksee (https://proksee.ca/projects), and the probable phage lifestyle (lytic or lysogenic) was predicted with Phage AI (https://accounts.phage.ai/). The amino acid sequences of the phage terminase large subunit were used to conduct phylogenetic trees of P40 and P49 using the one-click mode of Phylogeny (http://www.phylogeny.fr) [40].

We used Snippy v4.6.0 (https://github.com/tseemann/snippy) to identify SNPs between the genome sequence of the host strain and those of phage-resistant mutants. We used Roary v3.11.2 [41] and PIRATE v1.0.4 [42] to identify the insertion and deletion of genetic fragments in the phage-resistant mutants. Kaptive v2.0.0 (https://github.com/katholt/Kaptive) was used to type KL (capsule) [43]. The multiple sequence alignments of the *wcaJ*/*wbaZ*, *ugd*/*wbgU*, and *btuB* genes were performed using the Clustal Omega webserver (https://www.ebi.ac.uk/Tools/msa/clustalo/) [44]. Three-dimensional structure prediction was performed using SWISS-MODEL (https://swissmodel.expasy.org/) [45]. Then, root-mean-square deviation (RMSD) was calculated based on multiple structural alignments of BtuB using PyMOL (The PyMOL Molecular Graphics System, Version 3.0.3, Schrödinger, LLC). Values were reported in angstroms (Å). The primer pairs (Supplementary Table S4) were designed based on both ends of the deletion region, and PCR followed by Sanger sequencing was used to confirm genetic deletion. Insertion sequences were identified using ISfinder (https://www-is.biotoul.fr/index.php). According to the International Committee on Taxonomy of Viruses (ICTV), the species demarcation was defined as ≥95% overall DNA sequence similarity (identity × coverage) between two viral genomes was used to define species [46].

### Cloning and complementation experiments to study the phage-resistant mechanism

Cloning experiments were performed to identify which genes are responsible for phage resistance. The complete genomic sequence from the parental strain 135077 (GenBank accession no. CP073290) [26] was used as the template to amplify fragments of target genes using gene-specific primers (listed in Supplementary Table S4). Amplicons of the gene *ugd*, *wbgU, mdoD,* or *btuB* were digested using the corresponding restriction endonucleases and were then ligated with the cloning vector pBCSK (Stratagene; La Jolla, CA, USA) pre-treated with the same endonucleases. The recombinant plasmids containing two genetic components, namely *ugd* plus either *wbgU* or *wzm*/*wzt*, were assembled by a combination of seamless cloning (In-Fusion Snap Assembly Kit; Takara; Dalian, China) and restriction. The recombinant plasmids were then transformed into the corresponding phage-resistant mutants by heat shock. Transformants were screened on LB agar plates containing 50 μg/ml chloramphenicol at 37°C for 24 h, on which phages-resistant mutants cannot grow. Correct assembly of recombinant plasmids was verified by PCR using M13 forward and reverse primers, followed by Sanger sequencing. After complementation, the corresponding strain containing the recombinant plasmid was tested for susceptibility to phage P40 and/or P49 using the spot assay as described above, with phage-resistant mutants used as the negative controls.

### Knockout and complementation of the *ugd* and *wbgU* genes

To verify the role of the two genes in mediating phage P40 resistance alone, the construction of the *ugd*-deleted mutant and the *wbgU*-deleted mutant was performed in P04-resistant mutant PR1 strain using the CRISPR-Cas9-mediated genome-editing method containing pCasKP-apr (Addgene #117231) and pSGKP-km (Addgene #117233) plasmid [47]. Briefly, the *ugd*- and *wbgU*-specific spacer fragments were individually cloned into the pSGKP-apr vector. The donor templates were obtained by overlap extension PCR. Both the pSGKP-spacer plasmid and the donor template DNA were electroporated into the L-arabinose-induced pCasKP-harbouring PR1 cell. The cultures were plated on LB agar plates containing 30 μg/ml apramycin and 50 μg/ml kanamycin, and the colonies with successful recombination were screened by PCR and confirmed by Sanger sequencing. For plasmid curing, PR1Δ*ugd* and PR1Δ*wbgU* mutants were cultured on LB agar supplemented with 5% sucrose at 37°C. Chemically competent cells were prepared from PR1Δ*ugd* and PR1Δ*wbgU* strains. The plasmid pBCSK harbouring the *ugd* or *wbgU* gene was transformed into the corresponding competent cells using the heat shock method. All primers used in this study are listed in Supplementary Table S5.

### Statistical analysis

SPSS v23.0 (IBM; Armonk, NY, USA) was used for statistical analysis. All experiments were performed at least in duplicate (biological replicates). The details regarding the number of biological replicates are provided in the figure legends. OD values of bacteria, quantification of CPS and endotoxin were expressed as the mean ± standard deviation (SD). Continuous variables with a normal distribution were compared using Student’s *t*-test, while those with a non-normal distribution were analysed using the Mann–Whitney *U* test for inter-group differences. *p* < 0.05 was considered statistically significant.

## Results

### Recovery of P40, a new lytic phage against CRKP mutants resistant to P04, a previously isolated phage

**Figure 1.**
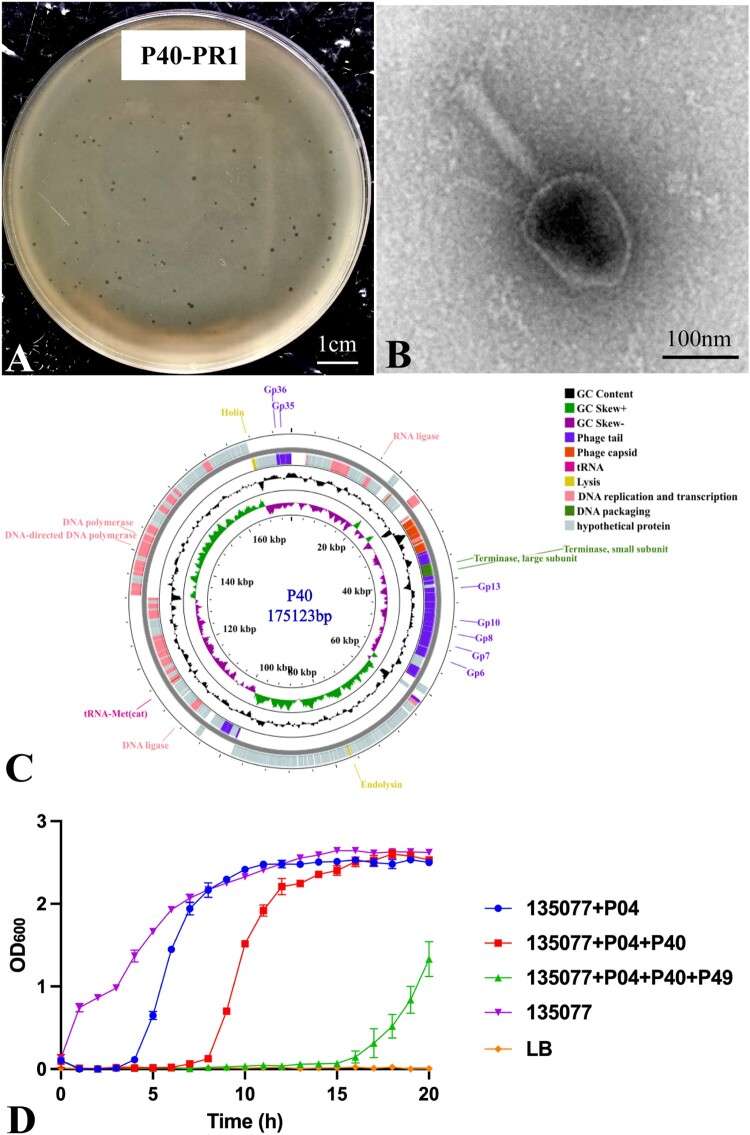
Biological and genomic characterization of phage P40. (A) P40 formed a circular plaque with ca. 1.0–2.0 mm (diameter). Scale bar, 1 cm. (B) The TEM image of P40. Scale bar, 100 nm. (C) Genome map of P40. Blocks in various colours represent predicted coding sequences (CDSs) encoding products of different functions. (D) The bacteriolytic activity of phage cocktail. Bacteriolytic activity of phages against CRKP strain 135077 at an MOI of 1. The values in both panels are the mean ± SD (*n* = 3).

We previously reported the isolation of a phage named P04 (also known as 150004), a member of the family *Autographiviridae*, which lysed an ST11-KL64 CRKP strain [26]. However, P04-resistant CRKP mutants emerged after a 4 h of exposure, resulting from the interruption of the CPS biosynthesis-associated gene *wcaJ* or *wbaZ* by insertion sequence IS*903B* alone or an IS*903B*-formed composite transposon [26]. To overcome the resistance, we used PR1 [26], a P04-resistant mutant of strain 135077, as the host bacterial strain and successfully isolated a new lytic phage, P40. P40 lysed PR1 efficiently, forming a clear plaque of a 1.0–2.0 mm diameter (Figure 1(A)), but did not lyse the original strain 135077.

In transmission electron microscopy (TEM), P40 displayed typical features of the long and contractile-tailed phage (Figure 1(B)), with a 100 nm icosahedral head and a 120 nm tail. The phage P40 is characterized by good stability, rapid adsorption to the host strain, and a large burst size of 112 ± 8 progeny phages per infected bacterial cell, indicating its strong lytic potential (Supplementary Figure S1). P40 has a double-stranded DNA (dsDNA) genome of 175,213 bp (Figure 1(C)). Compared to all sequenced phages on NCBI, P40 shared the highest nucleotide identity with *Slopekvirus* eap3 (accession no. NC_041980) with a 95.98% overall DNA sequence similarity (identity × coverage), which is above the 95% cutoff for the species demarcation defined by the ICTV [46]. Therefore, P40 belongs to the same species as *Slopekvirus* eap3, within the genus *Slopekvirus* of the family *Straboviridae* [48], as supported by a phylogenetic tree based on terminase large subunit sequences (Supplementary Figure S2). The P04 and P40 in combination could prolong growth retardation to nearly 8 h (Figure 1(D)). We then randomly collected three colonies (mutants) resistant to both P04 and P40, named B1E1, B1E2, and B1E3, respectively, for further study.

Recovery of phage P49 against P04/P40-resistant CRKP mutants, with cross-genus infection.

**Figure 2.**
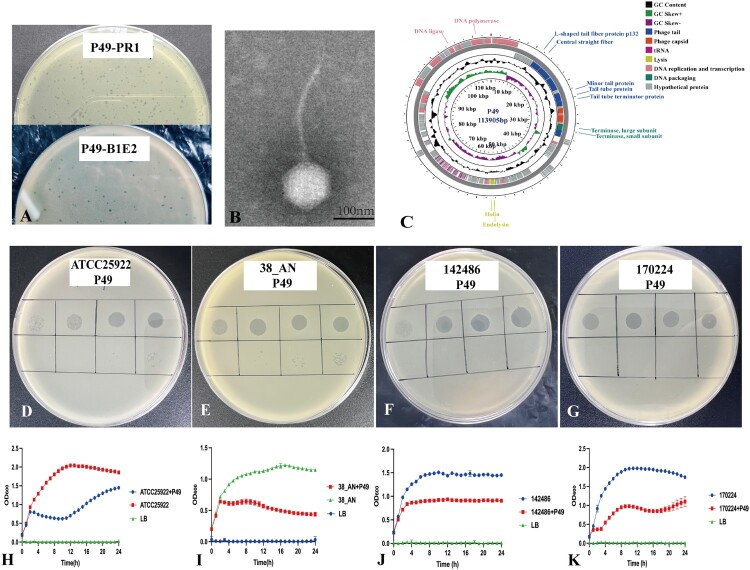
Biological and genomic characterization of phage P49. (A) P49 formed a circular plaque with ca. 1.0–2.0 mm (diameter) on B1E2 and PR1, respectively. Scale bar, 1 cm. (B) The TEM image of P49. P49 has an about 80 nm icosahedral head and an about 200 nm tail. Scale bar, 100 nm. (C) Genome map of P49. Blocks in various colours represent predicted CDSs encoding products of different functions. (D-G) The efficiency of plating assay confirmed that P49 could lyse *E. coli* ATCC25922, *S. enterica* 38_AN, *K*. *tianfuensis* 142486, and *E. ludwigii* 170224. (H-K) P49 could restrain the growth of *E. coli* ATCC25922 for 11 h, *S. enterica* 38_AN and *K*. *tianfuensis* 142486 for more than 24 h, and *E. ludwigii* 170224 for 16 h.

To overcome resistance to phages P04 and P40, a novel phage, P49, was isolated using P04/P40-resistant mutant B1E2 as the host bacterial strain. P49 formed clear plaques for P04-resistant mutant PR1 and P04/P40-resistant mutant B1E2 with plaque diameters ranging from 1.0 to 2.0 mm (Figure 2(A)), but it was unable to lyse the original strain 135077. TEM revealed typical features of the long and noncontractile-tailed phage (Figure 2(B)). The phage P49 also has good stability, rapid adsorption to host strain, and a high burst size of 157 ± 48 progeny phages per infected bacterial cell (Supplementary Figure S1). P49 has dsDNA of 113,905 bp (Figure 2(C)). Like P04 and P40, the P49 genome had no genes encoding antimicrobial resistance or virulence, meeting the criteria for phage therapy [49]. The combination of P04, P40, and P49 could restrain the growth of the original strain 135077 for 15 h (Figure 1(D)).

P49 had the highest nucleotide identity with *Epseptimavirus* 118970sal2 (accession no. KX017521) with a 92.04% overall DNA similarity, and P49 is a new species of the genus *Epseptimavirus*. Surprisingly, phage P49 is closely related to phages targeting *S. enterica* and *E. coli* in a phylogenetic tree inferred by amino acid sequences of the terminase large subunits (Supplementary Figure S3). We therefore assessed the host range of P49 against 7 *S. enterica* strains of six different serotypes and *E. coli* ATCC 25922 with P04 and P40 as controls. P49 was able to lyse *E. coli* ATCC25922, *E. ludwigii* 170224 (accession no. JBSATK01), *K*. *tianfuensis* 142486 (accession no. JBKHUI000000000), and *S. enterica* 38_AN (accession no. JBDLNS000000000) (Figure 2(D–H)). To further verify that P49 could infect bacteria from different genera, the phage bacteriolytic assay *in vitro* was performed. P49 could restrain the growth of *E. coli* ATCC25922 for 11 h, *E. ludwigii* 170224 for 16 h, and both *K*. *tianfuensis* 142486 and *S. enterica* 38_AN for more than 24 h (Figure 2(H–K)).

### The absence of UDP-glucose dehydrogenase-encoding *ugd* or NAD-dependent epimerase-encoding *wbgU* mediates resistance to phage P40

To elucidate the synergistic effects of the three phages, phages-resistant mutants were isolated. The P04-resistant mechanisms have been reported [26]. Therefore, we focused on the resistance to P40 and P49 in this study. The three P04- and P40-resistant (P04/P40-resistant) mutants were sequenced and compared with the original strain 135077. Except for an SNP of the *mdoD* gene encoding glucan synthesis-associated protein that was present in all three mutants compared with strain 135077, all other SNPs were nonsense or missense in genes encoding hypothetical proteins of unknown functions (Supplementary Table S6). By performing gene complementation and spot testing, we found that the susceptibility to P40 could not be restored by complementing the intact *mdoD* gene. This suggests that the mutation of *mdoD* is not responsible for resistance to P40.

**Figure 3.**
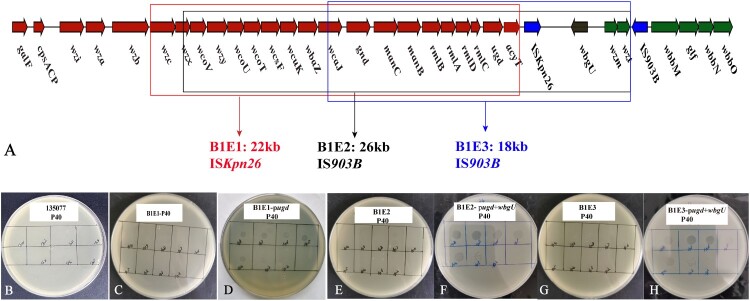
The lost genetic regions in P04/P40-resistant mutants and phage susceptibility tests. (A) The lost genetic regions in P04/P40-resistant mutants. Genes of the CPS biosynthesis gene cluster, genes of the O-antigen cluster, and insertion sequences are shown in red, green, and blue, respectively. The CPS biosynthesis gene cluster comprises *galF* (encoding UDP-glucose pyrophosphorylase), *cpsACP* (encoding acid phosphatase), *wzi* (related to capsule surface assembly), *wza* (encoding a CPS export protein), *wzb* (encoding tyrosine phosphatase), *wzc* (encoding tyrosine-protein kinase), *wzx* (encoding flippase), *wcoV* (encoding polysaccharide pyruvyl transferase), *wzy* (encoding an oligosaccharide-unit polymerase), *wcoU* (encoding UDP-Glc:α-D-GlcNAc-diphosphoundecaprenol β-1,3-glucosyltransferase), *wcoT* (encoding glycosyltransferase), *wcsF* (also called *mshA*, encoding glycosyltransferase), *wcuK* (encoding glycosyl hydrolase), *wbaZ* (encoding glycosyltransferase), *wcaJ* (encoding another glycosyltransferase), *gnd* (encoding gluconate-6-phosphate dehydrogenase), *manC* (encoding mannose-1-phosphate guanylyltransferase), *manB* (encoding phosphomannomutase/ phosphoglucomutase), *rmlB* (encoding dTDP-D-glucose-4,6-dehydratase), *rmlA* (encoding glucose-1-phosphate thymidylyltransferase), *rmlD* (encoding dTDP-6-deoxy-L-mannose dehydrogenase), *rmlC* (encoding dTDP-4-dehydrorhamnose reductase), *ugd* (encoding UDP-glucose dehydrogenase) and *acyT* (encoding acyltransferase). The O-antigen gene cluster contains *wzm* (encoding O-antigen synthesis transporter), *wzt* (encoding O-antigen synthesis transporter ATP-binding protein), *glf* (encoding UDP-galactopyranose mutase), *wbbM* (encoding glycosyltransferase), *wbbN* (encoding glycosyltransferase), and *wbbO* (encoding galactosyltransferase). The missing genetic regions are highlighted with boxes. (B) P40 could not lyse 135077. (C and D) B1E1 transformant after *ugd* gene complementation restored the sensitivity to P40. (E–H) B1E2 and B1E3 transformants after *ugd* and *wbgU* genes complementation restored the sensitivity to P40.

Insertion and deletion events in genetic regions were examined. All three mutants lost genetic components of varying size in the CPS biosynthesis gene cluster. B1E1 lost a 22-kb fragment between *wzc* (encoding a tyrosine-protein kinase) and *acyT* (encoding an acyltransferase; Figure 3(A)). B1E2 missed a 26-kb fragment between *wzx* (encoding a CPS flippase) and *wzt* (encoding the O-antigen synthesis ABC transporter ATP-binding protein, ca. 6.8 kb downstream of *ugd*) (Figure 3(A)). B1E3 had an 18-kb fragment absent between *wcaJ* (encoding a glycosyltransferase) and *wzt* (Figure 3(A)). Notably, the loss in all three mutants shared a fragment in common, which was a 12-kb region between *wcaJ* and *acyT* in the CPS biosynthesis gene cluster. The common missing fragment contains 10 genes, which have been reported to play a role in CPS biosynthesis [50,51]. Previously, we have identified that the absence of either *wbaZ* or *wcaJ* (encoding glycosyltransferase) mediates resistance to phage P04 [26]. *wcaJ* was absent from all three mutants and *wbaZ* was also absent in B1E1 and B1E2, explaining their resistance to P04. Next, we investigated the mechanisms mediating resistance to P40. P40 could not lyse 135077 (Figure 3(B)). Each of the 10 genes in the common missing fragment from strain 135077 was individually cloned into the three P04/P40-resistant mutants. Spot tests showed that B1E1 transformant containing *ugd* restored its susceptibility to P40 (Figure 3(C,D)), illustrating that the absence of *ugd* results in resistance to P40. However, surprisingly, the susceptibility to P40 was not restored in B1E2 and B1E3 when *ugd* was complemented (Supplementary Figure S4). This suggests the presence of additional mechanism (s) mediating resistance to P40 and indicates that *ugd* needs to act in combination with other gene (s) to confer susceptibility to P40.

Compared with B1E1, both B1E2 and B1E3 lost three additional genes, namely *wbgU* (encoding a NAD-dependent epimerase), *wzm* (encoding an O-antigen synthesis transporter), and *wzt* (encoding an O-antigen synthesis ABC transporter ATP-binding protein). The presence of *ugd* and *wbgU* together led to restoration of the susceptibility to P40 in both B1E2 and B1E3 (Figure 3(E–H)). The only complementation of *wbgU* did not restore the susceptibility to P40. All the above findings indicate that both *ugd* and *wbgU* are required for the susceptibility to P40, while the absence of any of the two genes or both could lead to resistance to this phage. P04/P40-resistant mutants after other genes complementation did not restore susceptibility to P40 (Supplementary Figure S5).

To verify the role of the two genes in mediating phage P40 resistance, PR1Δ*ugd* and PR1Δ*wbgU* strains were constructed. The two knockout mutants were resistant to P40, while the complemented strains restored the susceptibility to P40 (Supplementary Figure S6). The above results indicated that the alteration of either gene resulted in resistance to P40. Phage adsorption assays showed that P40 could not adsorb onto P04/P40-resistant mutants. P40 adsorbed P04/P40-resistant mutants after genes complementation rapidly (about 90% phages adsorbed at 20 min, Supplementary Figure S7A), indicating either of the two genes resulted in the loss or alteration of the phage receptor.

To uncover the mechanism responsible for the absence of large genetic fragments, PCR covering both ends of the missing fragments and sequencing the PCR amplicons were performed. In B1E1, B1E2, and B1E3, there was a single copy of IS*Kpn26*, IS*903B*, and IS*903B* replacing the missing fragment, respectively (Figure 3). There were no direct target repeats, which are characteristic of direct insertion. Therefore, the subsequent homologous recombination between the two copies of IS*Kpn26/*IS*903B* led to the deletion of the large fragments (Supplementary Figure S8–S10).

### The P40-resistance mechanism mediated by *ugd* and *wbgU* is associated with bacterial lipid A modification

**Figure 4.**
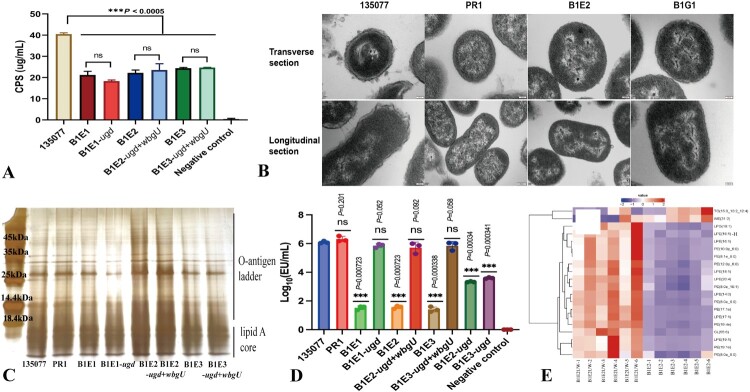
Characterization of CPS and LPS between P04/P40-resistant mutants and host strain. (A) Production of CPS. The CPS production of multi-phage-resistant mutants significantly decreased compared with that of host strain. (B) The TEM visualization of CPS. (C) The silver-stained gel of purified LPS. (D) The endotoxin levels. EU: endotoxin units. (E) Hierarchical clustering analysis of the differential lipids. The abscissa stands for samples, and the ordinate stands for different lipids. B1E2UW1-6 (abbreviated as B1E2UW-1, B1E2UW-2, B1E2UW-3, B1E2UW-4, B1E2UW-5, and B1E2UW-6) refers to six biological replicates of the B1E2 strain after genes complementation of *ugd* and *wbgU*. B1E21-6 (abbreviated as B1E2-1, B1E2-2, B1E2-3, B1E2-4, B1E2-5, and B1E2-6) refers to six biological replicates of the B1E2 strain. The first number in the brackets represents the number of carbon atoms, and the second one indicates the number of double bonds. e and p mean ether linkages and plasmalogen, respectively. TG, Triglyceride; WE, wax esters; LPG, lysophosphatidylglycerol; LPE, lysophosphatidylethanolamine; PE, phosphatidylethanolamine; CL, cardiolipin. Panels a, d, and f, data shown represent the mean ± SD of three independent experiments. Statistical significance was determined using Student’s *t* test. *p* values are shown in the figures: ns, *p* > 0.05; *, *p* ≤ 0.05; **, *p* ≤ 0.01; ***, and *p* ≤ 0.001.

As all P04/P40-resistant mutants missed some or all genes for CPS biosynthesis, we therefore measured the CPS production of phage-resistant mutants and transformants after genes complementation. The CPS production by the three P04/P40-resistant mutants ranged between 21.22 ± 1.70 µg/ml (mean ± SD) by B1E1 and 24.40 ± 0.36 µg/ml by B1E3, which were significantly less than that of strain 135077 (40.47 ± 0.65 µg/ml, *p* < 0.005). There were no differences in CPS amount between the three P04/P40-resistant mutants and transformants after genes complementation, indicating P49 does not recognize bacterial CPS (Figure 4(A)). In TEM (Figure 4(B)), strain 135077 has irregular and thickened CPS, 38 nm (mean) for the transverse section and 47 nm (mean) for the longitudinal one. By contrast, the thickness of CPS present on the bacterial surface was significantly reduced in P04-resistant mutant PR1 and the P04/P40-resistant mutant B1E2, <10 nm in both transverse and longitudinal sections, indicating a defect in CPS biosynthesis.

Therefore, we speculated that the P40 resistance mechanism was not mediated by CPS. In addition to CPS, both *ugd* and *wbgU* genes have been reported to be involved in LPS biosynthesis [52,53]. The silver-stained gel of LPS purified from the parental strain 135077, P04/P40-resistant mutants, and the corresponding strains with gene complementation exhibited the complete structure of LPS, including lipid A, core oligosaccharide, and an O-antigen in all strains (Figure 4(C)). This indicates that the alterations to the *ugd* or *wbgU* gene do not modify the structure of LPS.

Ugd, an UDP-glucose dehydrogenase, is involved in the modification of lipid A [54]. The alteration of lipid A can be demonstrated by measuring endotoxin [55,56]. The endotoxin level of P04/P40-resistant mutants (mean ± SD, ≤37.20 ± 8.18 EU/ml, Figure 4(D)) was remarkably lower than the P04-resistant PR1 ([1.55 ± 0.24] × 10^6^ EU/ml). There were no differences in the endotoxin level between P04/P40-resistant mutants after corresponding genes complementation and strain 135077. The endotoxin levels of B1E1 after complementing the intact *ugd* ([3.75 ± 1.09] × 10^5^ EU/ml, *p* > 0.05) recovered to the level of the parental strain 135077, suggesting that this gene is involved in the synthesis of endotoxin. The endotoxin level in B1E2 and B1E3 after complementing *ugd* only was not restored to the level of 135077, indicating that *wbgU* is also involved in endotoxin synthesis. Bacterial endotoxin is mainly dependent on lipid A [57]. Any modifications of lipid A, including the length and number of acyl chains and the number of phosphate groups, may lead to changes in endotoxin levels [56-58]. Therefore, the loss of either *ugd* or *wbgU* or both leads to resistance to P40 through modifications of bacterial lipid A.

Modifications to bacterial lipid A will affect bacterial membrane lipids [59,60]. To further investigate whether *ugd* and *wbgU* lead to modifications of bacterial lipid A, we performed untargeted lipidomic analysis. At the level of lipid subclass, lipid metabolites, including phosphatidylethanolamine (PE), lysophosphatidylethanolamine (LPE), phosphatidylcholine (PC), phosphatidylglycerol (PG), and lysophosphatidylglycerol (LPG) were significantly increased (*p* < 0.05) in the strain after complementing the intact *ugd* and *wbgU* together. We also performed cluster difference analysis at the lipid species level. The results showed that PE (16:0, 16:1, 17:1, 19:1; range, 1.8- to 5.8-fold, *p* < 0.05) and LPE (14:0, 16:1, 17:1, 18:1, 19:1, 20:4; range, 2.2- to 7.5-fold, *p* < 0.05) were significantly increased in the strain complemented by both *ugd* and *wbgU* (Figure 4(E)).

### P49 recognizes vitamin B_12_ transport protein-encoding gene *btuB*

**Figure 5.**
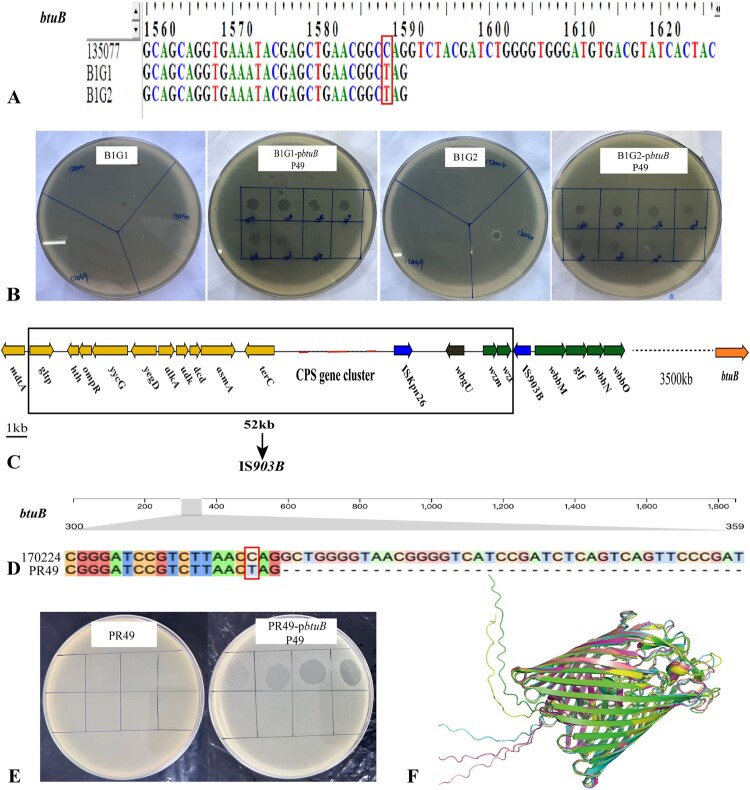
The mutation and deletion of genetic regions in P04/P40/P49-resistant mutants. (A) A SNP in gene *btuB* (encoding a vitamin B12 transport protein, shown in red) introduces a premature stop codon at the 1588th position in both B1G1 and B1G2. (B) Phage susceptibility tests. Transformants after the *btuB* gene complementation restored the sensitivity to P49. (C) P04/P40/P49-resistant B1G1 and B1G2 lost a 52-kb fragment between *gltP* and *wzt*. In addition to the CPS biosynthesis gene cluster, the missing fragment contains 10 genes involved in bacterial transcription and DNA repair. The 10 genes are *gltP* (encoding dicarboxylate/amino acid-cation symporter), *hth* (encoding response regulator transcription factor), *ompR* (encoding DNA-binding transcriptional dual regulator), *yycG* (encoding histidine kinase), *yegD* (encoding molecular chaperone), *alkA* (encoding DNA-3-methyladenine glycosylase 2), *udk* (encoding uridine kinase), *dcd* (encoding deoxycytidine triphosphate deaminase), *asmA* (encoding outer membrane assembly protein), and *terC* (encoding tellurium resistance protein). (D) A SNP in gene *btuB* (shown in red) introduces a premature stop codon at the 316th position in PR49. (E) Phage susceptibility tests. Transformants after the *btuB* gene complementation restored the sensitivity to P49. (F) The stereoscopic comparison of the three-dimensional structures of BtuB. Pink represents BtuB of ATCC25922. Yellow represents BtuB of 142486. Red represents BtuB of 170224. Blue represents BtuB of 38_AN. Green represents BtuB of 135077.

There was a SNP in the *btuB* gene (encoding a vitamin B_12_ transport protein) in the two P04/P40/P49-resistant mutants, named B1G1 and B1G2, which introduced a premature stop codon at the 1588th position (Figure 5(A), Table S6). B1G1 and B1G2 transformants containing wild-type *btuB* had restored susceptibility to P49 (Figure 5(B)). Phage adsorption assays showed that P49 could not adsorb onto P04/P40/P49-resistant mutants. About 90% of the P49 were adsorbed to P04/P40/P49-resistant mutants after *btuB* gene complementation at 20 min (Supplementary Figure 7B), indicating that vitamin B12 transport protein is the receptor of phage P49.

In addition, both B1G1 and B1G2 lost a 52-kb genetic fragment that contained the entire CPS biosynthesis gene cluster. This fragment was located between *gltP* (encoding a dicarboxylate/amino acid-cation symporter) and *wzt* (Figure 5(C)), and its deletion resulted in resistance to phages P04 and P40. Notably, this 52-kb fragment covers the 26-kb missing region in B1E2 and an extra 26-kb region containing six genes of the CPS biosynthesis gene cluster and 10 encoding bacterial transcription [61] or DNA repair [62]. We cloned each of the sixteen genes individually into B1G1 and B1G2, and none of the 16 genes mediated P49 resistance. Likewise, there was the homologous recombination between the two copies of IS*903B* resulting in the 52-kb large missing fragment (Supplementary Figure S11).

To further verify that the vitamin B_12_ transporter is the receptor recognized by P49, we cocultured strain 170224 with P49 and isolated a P49-resistant mutant named PR49. After sequence alignments, four SNPs were detected, one in the coding sequence (CDS) region and three in the untranslated region. There was an SNP in the *btuB* gene in PR49, which introduced a premature stop codon at the 316th nucleotide position (Figure 5(D)). Spot tests showed that the PR49 transformant containing *btuB* restored its susceptibility to P49, illustrating that P49 recognizes BtuB (Figure 5(E)). Based on the above experimental results and the cross-genus infection characteristics of P49, sequence alignment and structural analyses were performed. The pairwise sequence identity matrix (Supplementary Table S7) demonstrates significant similarity among sequences from distinct genera, with an overall average identity of approximately 68%. The 3D structures of the five BtuB proteins (Figure 5(F)) were further predicted using the SWISS-MODEL. The global RMSD values for all comparisons were below 0.65 Å (Supplementary Table S8), indicating that BtuB is highly conserved.

### Generalizability of phage cocktail resistance mechanisms in ST11-KL64 CRKP

To assess the generalizability of phage cocktail resistance mechanisms, we performed experiments by coculturing two distinct ST11-KL64 CRKP clinical isolates (strains 140494 and 140443) with a phage cocktail to isolate phage-resistant mutants. P04-resistant mutants (140494A1 and 140494A2) had mutations in the *wcaJ* gene encoding glycosyltransferase (Supplementary Table S6), and P04-resistant mutants (140443A1 and 140443A2) had interruptions by IS*Kpn14* in the *wcuK* gene encoding glycosyl hydrolase. P04/P40-resistant mutants (140494B1, 140494B2, 140443B1, and 140443B2) lost a large DNA fragment containing the *ugd* and *wbgU* genes (Supplementary Figure S12). In addition to large-fragment deletion (Supplementary Figure S12), P04/P40/P49-resistants (140494C1, 140494C2, 140443C1, and 140443C2) harboured mutations or interruptions by IS*Kpn26* in the *btuB* gene (Supplementary Table S6). In addition, pairwise sequence alignment revealed that the *wcaJ*, *wbaZ*, *ugd*, and *wbgU* genes shared 100% nucleotide identity among all ST11-KL64 CRKP strains examined, indicating that these genes are conserved (Supplementary Table S9). These results indicate that the resistance mechanisms of the phage cocktail, which include CPS deletion, *ugd*/*wbgU*-dependent lipid A change, and BtuB receptor alteration, are applicable to ST11-KL64 CRKP.

## Discussion

Phage therapy is a promising strategy for treating CRKP infection [63]. It is expected that the global phage market will reach 0.14 billion US dollars by 2028 [64]. There are two major approaches to constructing phage cocktails, namely commercial ones targeting multiple pathogens and personalized ones targeting specific pathogens [65]. In contrast to the many reports of successful cases of personalized phage therapy, randomized controlled trials of phage therapy have not brought the expected results due to the use of commercial phage products [66,67]. In this study, we constructed personalized phage cocktail using the “step-by-step” tandem approach. There was a distinct synergy among phages belonging to different families. The three phages exhibit stable biological characteristics and do not contain virulence factors or antimicrobial resistance genes [49], which represents a promising therapeutic option against CRKP.

To explore the stepwise mechanisms of synergy and phage resistance, we obtained three mutants resistant to both P04 and P40 and two mutants resistant to all three phages. Although P04/P40-resistant mutants lost a large fragment, the susceptibility to P40 could be restored by complementing the *ugd* and *wbgU* genes, and the absence of either gene or both leads to resistance to this phage. Previous studies have reported the loss of CPS large fragments containing *ugd* in phage-resistant strains targeting ST258 CRKP, but the specific gene mediating phage resistance has not been identified [18], indicating the generalizability of the finding in our study. In addition to CPS [68], the *ugd* is also involved in the synthesis of LPS [69]. Ugd, an UDP-glucose dehydrogenase, catalyzes the NAD-dependent transformation of UDP-glucose into UDP-glucuronic acid (UGA) [68]. UGA is converted into UDP-4-amino-4-deoxy-L-arabinose (L-Ara4N) to modify lipid A [52]. Lipid A works as a lipid anchor to the outer membrane and represents a central role for the immunological activity of endotoxin [70]. The endotoxin level in bacteria can be changed by lipid A modification, e.g. the size and number of lipid A acyl chains, the number of phosphate groups, and lipid architectures [56-58]. The *ugd* gene participates in the biosynthesis of L-Ara4N and further alters the lipid A structure. Therefore, the level of bacterial endotoxin was altered. *wbgU* has been reported to encode a UDP-N-acetylglucosamine (UDP-GlcNAc) C4 epimerase in *Plesiomonas shigelloides*, involved in the biosynthesis of LPS [53]. *wbgU* is located downstream of *ugd* and encodes the NAD-dependent epimerase involved in producing LPS, but its exact function in *K. pneumoniae* has not been reported. The modification of lipid A phosphates with L-Ara4N neutralizes the net negative charge on lipid A [56] and changes the hydrophobic effect of LPS [71], which can further remodel the bacterial outer membrane [72]. Previous studies have reported that PE and LPE are increased in *K. pneumoniae* possessing L-Ara4N-modified lipid A [60], which is consistent with our study. The deletion of the *ugd*/*wbgU* genes affects the modification of bacterial lipid A, thereby altering the membrane lipid composition. This change impacts the accessibility of an unidentified membrane protein for phage adsorption. Therefore, we speculated that P40 recognizes bacterial lipid A or an unidentified membrane protein.

Insertion sequences are ubiquitous in bacterial genomes and have been well documented to enable generating genetic plasticity to mediate antimicrobial resistance [73] and phage resistance [74]. The loss of large genetic fragments in bacterial mutants resistant to phages could be considered more unusual. Intriguingly, IS*903* has the unusual ability to transpose in both non-replicative (cut-and-paste, also called simple insertion) and replicative (copy-and-paste, generating a cointegrate) pathways [75,76]. It is therefore reasonable to believe that IS*903B* can behave like IS*903* to cause adjacent deletions via intramolecular replicative transposition, which may explain the loss of large genetic fragments seen in this study. This may represent a generalized anti-phage mechanism for bacteria.

BtuB is an outer membrane protein involved in the transport of vitamin B_12_ [77,78]. BtuB crosses the outer membrane, which is an asymmetric bilayer consisting of phospholipids and LPS [79,80]. The conserved BtuB structure provides a solid molecular basis for the observed “rare cross-genus infection” by phage P49. This is supported by its identification as a receptor for multiple phages infecting *S. enterica* and *E. coli* [81-83], which accounts for the lytic activity of P49 against these bacterial genera. However, successful infection of phages requires not only a compatible receptor but also its physical accessibility on the bacterial surface. P49 can lyse the CPS-deficient strains PR1 and B1E2, but cannot lyse the parental strain 135077, demonstrating that CPS loss exposes BtuB. This mechanism explains the observed infection patterns: BtuB is naturally available to bacteria that are not encapsulated, like *S. enterica*. Therefore, successful cross-genus infection by phage P49 is determined not only by the conserved structure of the BtuB but also by its accessibility. The observed infection pattern reflects a permissive combination of conserved receptor architecture and a bacterial surface that renders it accessible.

A striking finding worth highlighting is that P49 can lyse several other *Enterobacteriaceae* species such as *S. enterica* and *E. coli*, indicating that bacterial mutants resistant to one type of phages will expose originally covered targets vulnerable to the attack of other types of phages, including those primarily preying on other closely related bacterial species. This expands the bank of potential phages against the target bacterial species or strains. The cross-over activities of phages against wild-type strains of one bacterial species and mutant strains of another species also raise fundamental questions about the host range of phages. The terms such as “narrow host range”, “species-specific”, and “strain-specific” may require more cautious usage as the described phages may exhibit activities against certain mutants of other strains and/or species.

We are aware of the limitations of this study. First, although we found that the alteration of either *ugd* or *wbgU* or both mediated resistance to phage P40 by lipid A modification, the exact mechanism of lipid A modification is yet to be determined. Second, the number of P49-susceptible strains available for study is relatively limited, preventing the expansion of our conclusions to a broader context. The accessibility of BtuB is closely associated with bacterial surface structures, such as CPS and exopolysaccharides. Therefore, the inability of P49 to lyse a particular strain cannot be definitively attributed to a failure to recognize BtuB, as the receptor may be physically shielded. This inherent confounding factor makes direct structure comparison of BtuB between lysed and non-lysed strains infeasible. Third, we must also now expand this work beyond the single ST11-KL64 CRKP studied. Whilst this is the predominant type of CRKP in China, there is significant geographical variation in dominant CRKP lineages. The three-phage scheme here is unlikely to serve as a one-size-fits-all solution elsewhere but creates a vital blueprint for further studies across the species and other multi-drug resistant Gram-negative pathogens. Consequently, phage cocktail resistance mechanisms will be verified beyond ST11-KL64, which represents a critical future direction. This will entail validating the resistance mechanisms in other genetic backgrounds and capsular types, coupled with direct chemical analysis of lipid A variants and quantitative assessment of BtuB surface exposure.

In conclusion, we created a combination of three stepwise recovered lytic phages targeting bacterial CPS, lipid A or unidentified protein, and BtuB, respectively, which provides a promising therapeutic option. In particular, P49 could lyse several other *Enterobacteriaceae* such as *S. enterica and E. coli*, which allows for screening more phages that are originally thought to be ineffective and therefore may create new cocktails to overcome resistance to previously used phages, opening a new world for creating phage cocktails.

## Author contributions

X.Y. and Z.Z. conceived and designed the study. A.M. involved in the study design. X.Y. performed the experiments, analysed the data, and drafted the manuscript. H.L. participated in *in vitro* phage experiments. J.F. participated in phage assays against *Salmonella*. Y.F., Q.F, and Z.Z. participated in data analysis. Z.Z. and A.M. revised the manuscript. All authors have read and approved the manuscript.

## Supplementary Material

Supplementary material clean.docx

## Data Availability

The complete sequence of phage P40 and the complete genome of phage P49 have been deposited in GenBank under accession numbers OP045497 and OP045498, respectively. The accession numbers of other phage-resistant mutants in our study can be found in Supplementary Table S10. All other data are in the manuscript and its supplementary file.
