# Supplementary material for: Stepwise phage resistance and collateral phage susceptibility in Klebsiella pneumoniae
Source: Emerg Microbes Infect. 2026 Mar 19;15(1):2648890. doi: 10.1080/22221751.2026.2648890 (PMC13224724; doi:10.1080/22221751.2026.2648890)

**Supplementary material**

Supplementary Text S1. The supplementary materials and methods.

Supplementary Table S1. The six intact prophage regions identified in CRKP strain 135077.

Supplementary Table S2. CRKP used in this study and the host range of phages.

Supplementary Table S3. Strains of the family *Enterobacteriaceae* used for determining the host range of phages.

Supplementary Table S4. Primers used for amplification and sequencing.

Supplementary Table S5. The primers for gene knockout and validation.

Supplementary Table S6. SNPs between the genome sequence of host strain and phage-resistant mutants.

Supplementary Table S7. Supplementary Table S7. Pairwise nucleotide sequence identity (%) matrix of the *btuB* gene from cross-genus strains.

Supplementary Table S8. Results of global structural alignment of BtuB homologs.

Supplementary Table S9. Pairwise nucleotide sequence identity (%) matrix of *wcaJ*, *wbaZ*, *ugd*, and *wbgU* genes from ST11-KL64 CRKP in our study.

Supplementary Table S10. The accession numbers of other phage-resistant mutants in our study.

Supplementary Figure S1. Biological characterization of P40 and P49.

Supplementary Figure S2. The phylogenetic tree of phage P40.

Supplementary Figure S3. The phylogenetic tree of phage P49.

Supplementary Figure S4. Phage P40 susceptibility tests targeting B1E2 and B1E3 complemented with *ugd* and *wbgU* alone.

Supplementary Figure S5. Phage P40 susceptibility tests targeting P04/P40-resistant mutants after gene complementation.

Supplementary Figure S6. Phage P40 susceptibility tests targeting P04/P40-resistant mutants after gene knockout and complementation.

Supplementary Figure S7. Phage adsorption rate.

Supplementary Figure S8. The homologous recombination of two copies of IS*Kpn26* resulted in the loss of the 22-kb fragment in B1E1.

Supplementary Figure S9. The homologous recombination of two copies of IS*903B* resulted in the loss of the 26-kb fragment in B1E2.

Supplementary Figure S10. The homologous recombination of two copies of IS*903B* resulted in the loss of the 18-kb fragment in B1E3.

Supplementary Figure S11. The homologous recombination of two copies of IS*903B* resulted in the loss of the 52-kb fragment in B1G1 and B1G2.

Supplementary Figure S12. The lost genetic regions in P04/P40-resistant mutants and P04/P40/P49-resistant mutants were identified by coculturing the other ST11-KL64 CRKP with the phage cocktail.

**Supplementary Text S1. The supplementary materials and methods.**

**The optimal multiplicity of infection and adsorption rate of phage**

The optimal multiplicity of infection (MOI) refers to the ratio between phage and host bacterial strain that generates the most phage progenies^1^. The host strain was cultured until the logarithmic growth phase in LB broth was reached and was then adjusted to 0.5 McFarland (about 10^8^ colony forming units [CFU]/ml). An equal volume of phage suspensions at series titers (10^4^ to 10^9^ PFU/ml) was added to the bacterial broth and was then incubated at 37 °C with 180 rpm vortex for 5 min. The co-cultures were centrifuged for 3 min at 13,000 rpm, then resuspended in fresh LB broth to remove unabsorbed phages. The supernatant was transferred into sterile Eppendorf (EP) tubes and used to determine the number of unabsorbed phages. Incubation was continued for 4 h at 37 °C, and the titer of phage at different MOI was tested using the double-layer agar method. The MOI with the highest phage titer was defined as the optimal MOI.

Phage adsorption assay^2^ reflects the time for the phages to adsorb to the host strain. Phage was added to the host bacterial suspension (0.5 McFarland) at the optimal MOI and incubated at 37 °C and 180 rpm. At 0, 3, 6, 9, 12, 15 min, and 20 min, 1 ml aliquots were retrieved to count phage titers. The phage adsorption rate was calculated as follows: adsorption rate (%) = [ (initial phage number − phage number in the supernatant) / (initial phage number)] × 100^3^. The experiments were performed in triplicate.

**One-step growth curve of phages**

To determine the burst sizes and latent periods of phages, we performed the one-step growth curve of phages using a standard method as described previously^4^. In brief, 100 µl of phage was added with the host strain (10^8^ CFU/ml) to reach the optimal MOI, and then the co-culture was incubated at 37 °C for 5 min. Subsequently, the co-culture was diluted in tubes A, B, and C by 100, 1,000, and 10,000 times, followed by incubation at 37 °C. In addition, a 1-ml aliquot from tube A was filtered and used to count unabsorbed phages. Aliquots (100 μl) were retrieved at a 2-min interval for 120 min for counting the number of phages using the double-layer plate assay^5^. The burst size of phages was calculated using the formula: burst size = (the number of phages at the end of lysis – the number of unabsorbed phages) / the initial number of infected bacterial cells^6^. The experiments were performed in triplicate.

**pH and thermal stability of phages**

The stability of phages at different pH values and temperatures determines the mode of administration and storage conditions. For the pH stability test, the phage suspension was added to TM buffer with different pH values (1 to 13) to a final concentration of 10^8^ PFU/ml. The culture was incubated at 37 °C for 1 h. The thermal stability of the phage was tested at 4, 25, 37, 50, 60, 70, and 80 °C for 1 h in a water bath. The phage titer was determined using the double-layer agar method^5^. The experiments were performed in triplicate.

**TEM to observe morphology of phages and bacteria**

Phage particles were precipitated in NaCl and PEG8000, collected by centrifugation at 4,700 rpm for 20 min in a 4 °C pre-cold centrifuge, and resuspended in TM buffer^7^. The phage particles were dropped on carbon film-coated copper grids (Carbon Type-B 100 mesh; Zhongjingkeyi, Beijing, China) for 10 min, and then a drop of 2% (w/v) uranyl acetate was added for negative staining. The morphology and size of the phage were observed using a JEM-1400 PLUS TEM (JEOL, Tokyo, Japan) at an accelerating voltage of 80 kV with a minimum resolution of 10 nm.

**The phage-resistant mutants isolation**

Briefly, 10 µl phage stock solution at 10^8^ PFU/ml and 100 µl host bacterial strain at the logarithmic growth phase were added to 5 ml of 0.7% molten top LB agar and poured over LB plates. After overnight incubation at 37 °C, individual colonies were picked into LB broth at 180 rpm for 4-6 h to the logarithmic growth phase. The susceptibility of individual colonies to phages was determined by pipetting 3 µl phage stock solution dropped onto the lawns of bacterial cells overlayed on LB plates. The phage-resistant mutants were further purified by streaking onto LB plates three consecutive times, and the resistance to phages was further confirmed by spot testing. All strains were archived in 20% glycerol and stored at -80 °C for further analysis.

**References**

1 Abedon, S. T. & Katsaounis, T. I. Basic phage mathematics. *Methods Mol Biol* **1681**, 3-30, doi:10.1007/978-1-4939-7343-9_1 (2018).

2 Kropinski, A. M. Measurement of the rate of attachment of bacteriophage to cells. *Methods Mol Biol* **501**, 151-155, doi:10.1007/978-1-60327-164-6_15 (2009).

3 Duarte, A. C. *et al.* Synergistic action of phage phiIPLA-RODI and lytic protein CHAPSH3b: a combination strategy to target *Staphylococcus aureus* biofilms. *NPJ Biofilms Microbiomes* **7**, 39, doi:10.1038/s41522-021-00208-5 (2021).

4 Kropinski, A. M. Practical advice on the one-step growth curve. *Methods Mol Biol* **1681**, 41-47, doi:10.1007/978-1-4939-7343-9_3 (2018).

5 Van Twest, R. & Kropinski, A. M. Bacteriophage enrichment from water and soil. *Methods Mol Biol* **501**, 15-21, doi:10.1007/978-1-60327-164-6_2 (2009).

6 Sattar, S. *et al.* Genome analysis and therapeutic evaluation of a novel lytic bacteriophage of *Salmonella Typhimurium*: suggestive of a new genus in the subfamily *Vequintavirinae*. *Viruses* **14**, 241, doi:10.3390/v14020241 (2022).

7 Ackermann, H.-W. Basic phage electron microscopy. *Methods Mol Biol* **501**, 113-126, doi:10.1007/978-1-60327-164-6_12 (2009).

Supplementary Table S1. The six intact prophage regions identified in CRKP strain 135077.

| Region | Region length | Completeness (score) | Specific keyword | Position |
| --- | --- | --- | --- | --- |
| 1 | 61.3 kb | Intact (118) | plate, lysin, tail | chr: 1219040-1280356 |
| 2 | 53.9 kb | Intact (150) | integrase, tail, terminase, protease, capsid, plate, lysin, lysis | chr: 1372900-1426804 |
| 3 | 56.8 kb | Intact (110) | plate, lysin, tail, head, portal, terminase, integrase | chr: 3029415-3086246 |
| 4 | 40.3 kb | Intact (110) | tail, capsid, head, portal, terminase, lysis | chr: 3294401-3334717 |
| 5 | 37.1 kb | Intact (141) | capsid, lysin | chr: 3461440-3498546 |
| 6 | 31.5 kb | Intact (119) | lysin, plate | chr: 3659536-3691035 |

Supplementary Table S2. CRKP used in this study and the host range of phages

| **Strains** | **ST** | **Capsule type** | **Carbapene-**  **mase genes** | **Source** | **Accession no.** | **Sensibility to** | | |
| --- | --- | --- | --- | --- | --- | --- | --- | --- |
|  |  |  |  |  |  | **P04** | **P40** | **P49** |
| 135040 | ST11 | KL47 | *bla*_KPC-2_ | sputum | JANHBU000000000 | - | - | - |
| 135042 | ST11 | KL47 | *bla*_KPC-2_ | sputum | JANHBT000000000 | - | - | - |
| 135079 | ST11 | KL47 | *bla*_KPC-2_ | secretion | JANHBQ000000000 | - | - | - |
| 135044 | ST11 | KL64 | *bla*_KPC-2_ | urine | JANHBS000000000 | + | - | - |
| 135055 | ST11 | KL64 | *bla*_KPC-2_ | sputum | JANHBR000000000 | + | - | - |
| 135077 | ST11 | KL64 | / | urine | CP073290-CP073296 | + | - | - |
| 135025 | ST11 | KL64 | *bla*_KPC-2_ | section | JANHBV000000000 | + | - | - |
| 135080 | ST11 | KL64 | *bla*_KPC-2_ | urine | JANHBP000000000 | + | - | - |
| 130018 | ST11 | KL64 | *bla*_KPC-2_ | blood | JANHBW000000000 | + | - | - |
| 140077 | ST11 | KL64 | *bla*_KPC-2_ | blood | JANHBO000000000 | + | - | - |
| 015134 | ST11 | KL64 | *bla*_KPC-2_ | anal swab | JBUYSP000000000 | + | - | - |
| 140531 | ST11 | KL64 | *bla*_KPC-2_ | ascite | JBUYSM000000000 | + | - | - |
| 140494 | ST11 | KL64 | *bla*_KPC-2_ | sputum | JBUYSN000000000 | + | - | - |
| 140443 | ST11 | KL64 | *bla*_KPC-2_ | urine | JBUYSO000000000 | + | - | - |
| 115057 | ST11 | KL39 | *bla*_OXA-181_ | sputum | JANHBK000000000 | - | - | - |
| 140127 | ST11 | KL21 | *bla*_KPC-2_ | blood | JANHBN000000000 | - | - | - |
| 140191 | ST11 | KL25 | *bla*_KPC-2_ | blood | JANHBM000000000 | - | - | - |
| 170094 | ST11 | KL25 | *bla*_KPC-2_ | secretion | JBEVTL000000000 | - | - | - |
| 020035 | ST45 | KL62 | *bla*_KPC-2_ | secretion | NIVL00000000 | - | - | - |
| 020116 | ST307 | KL102 | *bla*_NDM-1_ | blood | PWCX00000000 | - | - | - |
| 141260 | ST147 | KL10 | *bla*_KPC-2_ | blood | JBEFAT000000000 | - | - | - |
| 170047 | ST15 | KL19 | *bla*_KPC-2_ | BALF | JBEFAO000000000 | - | - | - |
| 090571 | ST1537 | KL24 | *bla*_NDM-1_ | sputum | JBEFBE000000000 | - | - | - |
| 141514 | ST307 | KL102 | *bla*_NDM-5_ | sputum | JBEFAR000000000 | - | - | - |
| 090223 | ST37 | KL28 | *bla*_KPC-2_ | blood | JBEFBH000000000 | - | - | - |
| 020136 | ST1 | KL45 | *bla*_KPC-2_ | blood | NWFF000000000 | - | - | - |
| 141578 | ST16 | KL51 | *bla*_OXA-48_ | sputum | JBEFAQ000000000 | - | - | - |
| 020117 | ST45 | KL52 | *bla*_NDM-1_ | sputum | PWAI000000000 | - | - | - |
| 140179 | ST101 | KL106 | *bla*_NDM-1_ | sputum | JBEFAZ000000000 | - | - | - |
| 090107 | ST16 | KL51 | *bla*_NDM-5_ | blood | VCBI00000000 | - | - | - |
| 090566 | ST789 | KL18 | *bla*_NDM-5_ | blood | JACWGL000000000 | - | - | - |
| 140529 | ST709 | KL9 | *bla*_KPC-2_ | secretion | JANHBL000000000 | - | - | - |
| PR1 | */* | */* | */* | */* | CP101726-CP101730 | - | + | + |
| B1E1 | */* | */* | */* | */* | JAWQTE000000000 | - | - | + |
| B1E2 | */* | */* | */* | */* | JAWQTF000000000 | - | - | + |
| B1E3 | */* | */* | */* | */* | JAWQTG000000000 | - | - | + |
| B1G1 | */* | */* | */* | */* | JAWQTH000000000 | - | - | - |
| B1G2 | */* | */* | */* | */* | JAWQTI000000000 | - | - | - |

Note: “-” represents that the bacteria could not be infected by the phages. “+” represents that the bacteria could be infected by the phages. “/” represent phages resistant mutants and does not contain the information.

Supplementary Table S3. Strains of the family *Enterobacteriaceae* used for determining the host range of phages.

| **Strain** | **Serotype/Species** | **Accession no.** | **Sensibility to P04** | **Sensibility to P40** | **Sensibility to P49** |
| --- | --- | --- | --- | --- | --- |
| 38_AN | Typhimurium | JBDLNS000000000 | - | - | + |
| ATCC 13076 | Enteritidis | / | - | - | +- |
| 14 | Enteritidis | / | - | - | - |
| SE1 | Derby | / | - | - | - |
| ATCC 14028 | Typhimurium | / | - | - | - |
| AH6 | Indiana | / | - | - | - |
| B.6 | Thompson | / | - | - | - |
| ATCC25922 | *Escherichia coli* | / | - | - | + |
| 035187 | *Escherichia coli* | JBSATI01 | - | - | - |
| 035190 | *Escherichia coli* | JBSATH01 | - | - | - |
| 170224 | *Enterobacter ludwigii* | JBSATK01 | - | - | + |
| 170186 | *Enterobacter sichuanensis* | JAVISB01 | - | - | - |
| 155091 | *Enterobacter xiangfangensis* | JBSATL01 | - | - | +- |
| 170236 | *Enterobacter* *hoffmannii* | JBSATJ01 | - | - | - |
| 120115 | *Enterobacter* *hoffmannii* | JAHEVS01 | - | - | +- |
| 090027 | *Enterobacter* *hormaechei* | RXRJ01 | - | - | - |
| 090019 | *Enterobacter hoffmannii* | RXRC01 | - | - | - |
| 090309 | *Enterobacter hoffmannii* | JAHESK01 | - | - | - |
| 142486 | *Kluyvera tianfuensis* | JBKHUI000000000 | - | - | + |
| 142212 | *Kluyvera cryocrescens* | SRR35926722 | - | - | - |

Note: “-” represents that the bacteria could not be infected by the phages. “+” represents that the bacterial strain could be infected by the phages. “+-” represents that the bacterial strain could be adsorbed by the phage but could not be infected by it. “/” does not contain this information.

Supplementary Table S4. Primers used for amplification and sequencing.

| **Primer** | **Amplified genes** | **Sequence (5′→3′)** | **Restriction enzyme** |
| --- | --- | --- | --- |
| *mdoD*-CF | *mdoD* | GACTAGTCGAGGAGCGATACATGG | *Spe*I |
| *mdoD*-CR |  | AACGAGCTCGTACTGCTAGGATATCT | *Sac*I |
| *ugd*-CF | *ugd* | GGACTAGTAACGATCCGGAGATAGG | *Spe*I |
| *ugd*-CR |  | TCCCCGCGGGGCATCTAATATATTTA | *Sac*I |
| *btuB*-CF | *btuB* | AACGGATCCGTCGCACTGGGTTTTAT | *BamH*I |
| *btuB*-CR |  | AACGAGCTCGTAATGCAGATTCGGCA | *Sac*I |
| *wbgU*-CF | *wbgU* | CCCAAGCTTGTCTTTCGCGAATGATA | *Hind*III |
| *wbgU*-CR |  | AACGGATCCGCGAGTATAAAGGCAAA | *BamH*I |
| *galf*-CF | *galF* and *cpsACP* | CGGGATCCCGAGCCGCTGAATAACCTGAA | *BamH*I |
| *ACP*-CR |  | CGAGCTCGGTCCTTTCTCAGCGCATT | *Sac*I |
| *wzi*-CF | *wzi* | GGAATTCTGCCGCGAGCGCTTTCTATC | *EcoR* I |
| *wzi*-CR |  | GCTCTAGAAAGAGCTGACTGCATCCTCCA | *Xba* I |
| *wza*-CF | *wza* | CGGGATCCCTCTCCAAAGCCGGCAA | *BamH*I |
| *wza-CR* |  | CGAGCTCACCCGCTAGCGAGAGATCG | *Sac*I |
| *wzb*-CF | *wzb* | CGGGATCCGACGAAGCATTCGCATCTGT | *BamH*I |
| *wzb*-CR |  | GGAGCTCCAATGTTGATATGCCCCCTTTTG | *Sac*I |
| *wzc*-CF | *wzc* | CGGGATCCCAAGTAGTTACTATAGTTACGGG | *BamH*I |
| *wzc*-CR |  | CCGAGCTCACAGGTAGCCCCGATATT | *Sac*I |
| *terC*-CF | *terC* | CCCAAGCTTATGGAATGGATTGCCGATCCG | *Hind*III |
| *terC*-CR |  | TCCCCGCGGTTACACCTCATAATCCAGCTCATC | *Sac* II |
| *asma*-CF | *asmA* | CCGCTCGAGATAACGTCAACGGGGTGTTG | *Xho* I |
| *asma*-CR |  | CGGGATCCCCTACATTAAGTGGCGACGC | *BamH*I |
| *alka*-CF | *alkA*, *udk*, and *dcd* | CCCAAGCTTATGGTGCTGTTACCCTGGAC | *Hind*III |
| *dcd*-CR |  | GCTCTAGAGTCAGTATTCGTCTCATGGCAC | *Xba* I |
| *yegd*-CF | *yegD* | CCCAAGCTTGCGACGATCCGCTAAAGTC | *Hind*III |
| *yegd*-CR |  | GCTCTAGAAACAGGCCATTTTGCGTGG | *Xba* I |
| *yycg*-CF | *yycG* | CCCAAGCTTAATGGCCTGTTCGTCCGGA | *Hind*III |
| *yycg*-CR |  | CCGGATCCCGGCGTTATCTTCAATTATCGC | *BamH*I |
| *ompR*-CF | *ompR* and *hth* | CCCAAGCTTTATTCTCACCCTCCCCAC | *Hind*III |
| *hth*-CR |  | GCTCTAGACTACCAGCGCTCACGTAA | *Xba* I |
| *gltp*-CF | *gltP* | GGGGTACCGTGGCAAACGCAAACAAACTC | *Kpn* I |
| *gltp*-CR |  | GCTCTAGATCAGGCTTCCGCTTTCTCCA | *Xba* I |
| *acyT*-CF | *acyT* | CCGCTCGAGATGAGAGAAACATGGGTTGAC | *Xho* I |
| *acyT*-CR |  | GCTCTAGATCATATCAGGAACTTGTTCGCACCTTCCCTAAGT | *Xba* I |
| *wcaJ-CF* | *wcaJ* | CCCAAGCTTGGATAATTTCATTAATG | *Hind*III |
| *wcaJ-CR* |  | CGGGATCCCTGAACACATTACTAAC | *BamH*I |
| *gnd*-CF | *gnd* | CGGGATCCGTGTTCAGAATGTGCAT | *BamH*I |
| *gnd*-CR |  | CCCAAGCTTGCAGATTAATATAGGCGTAC | *Hind*III |
| *manC*-CF | *manC* | CGGGATCCTGACCTTAAGCATTTC | *BamH*I |
| *manC*-CR |  | AACAAGCTTTCACCACGGATGTCATA | *Hind*III |
| *manB*-CF | *manB* | CGGGATCCTCGTATTAAAGACCAGTATG | *BamH*I |
| *manB*-CR |  | CCCAAGCTTCAAATAGTCGTTTCATTAA | *Hind*III |
| *rmlB*-CF | *rmlB* | CGGGATCCGAGAAGAAAACAATAGAA | *BamH*I |
| *rmlB*-CR |  | CCCAAGCTTCATCTTTACTTCCTTATTAG | *Hind*III |
| *rmlA*-CF | *rmlA* | CTATCTAGATCCTATGCCGGTGAGCGT | *Xba* I |
| *rmlA*-CR |  | AACGAGCTCTCAAAGTAATCCACCGC | *Sac*I |
| *rmlD*-CF | *rmlD* | CGGGATCCATAACAAGATGAAGATATT | *BamH*I |
| *rmlD*-CR |  | AACAAGCTTGCTACCCGTTTATTTAC | *Hind*III |
| *rmlC*-CF | *rmlC* | GCTCTAGAATTCCAGCAGGCATTTG | *Xba* I |
| *rmlC*-CR |  | AACGAGCTCTTAAATAATGCTGTGCG | *Sac*I |
| *ugd*-F | *ugd* | GCAGCCCGGGGGATCAAGTTTATAAATAATTAG | - |
| *ugd*-R |  | AAACAATGATGAGGAATTGAAAAAATACTACAAACC | - |
| *wbgU*-F | *wbgU* | TCCTCATCATTGTTTATATTGGGTTTTATATTATTTCAACTT | - |
| *wbgU*-R |  | GGAACAAAAGCTGGAAAAATAATGAATGTGCATTC | - |
| *ugd*-FF | *ugd* | CGGTATCGATAAGCTAAGTTTATAAATAATTAG GTTGTTTTTCCTAAAATGGCGG | - |
| *ugd*-FR |  | AAACAATGATGAGGAATTGAAAAAATACTACAA ACCCTTATCTCC | - |
| *wzmt*-FF | *wzm* and *wzt* | TCCTCATACCTGATAAACTTTGCCCGCAG | - |
| *wzmt*-RR |  | GGGAACAAAAGCTGGGCTGGTATAGATTTTAAC GCTATTGTTCAT | - |
| M13-IF* | - | CAGGAAACAGCTATGACC | - |
| M13-IR* | - | GTAAAACGACGGCCAGT | - |
| B1E1-F# | - | AGCCCGAACGCAGGTAAG | - |
| B1E1-R# | - | GCTTCAGCCTCAAGTAGTTAC | - |
| B1E2-F# | - | CGGATGTTACCCTATAATAGGCG | - |
| B1E2-R# | - | ATACCTTCGACACACCGTTG | - |
| B1E3-F# | - | GTGTGTGTTTTCAGTGGGGTT | - |
| B1E3-R# | - | ATACCTTCGACACACCGTTG | - |
| B1G1-F# | - | TGCCAGAACCAGTAGGCGG | - |
| B1G1-R# | - | ATACCTTCGACACACCGTTG | - |
| 224B-F | btuB | CCCCCCCTCGAGGTCGACTGAAGCCTGCGGCATCCT | - |
| 224B-R |  | CCCGGGCTGCAGGAATTCTTAGAAGGTGTAGCTGCCAGACAAG | - |

Note: The seamless cloning primers are underlined. The primers used for sequencing were annotated with an asterisk. The primers designed at both ends of the lost gene fragments are marked with #.

Supplementary Table S5. The primers for gene knockout and validation.

| **Primer** | **Sequence (5′→3′)** | **Primer use** |
| --- | --- | --- |
| ugd-spacer-F | TAGTCTGGTTAAGCATATCCACTT | *ugd* spacer for gene deletion and replacement |
| ugd-spacer-R | AAACAAGTGGATATGCTTAACCAG |  |
| ugd-up-F | GAGAGCTTCAGCAGCAAGGTG | amplification of *ugd* upstream locus from genome |
| ugd-up-R | CGCCTTCTTTCAGCAGGTCATGCGCAATCAGGACACCGTT |  |
| ugd-down-F | AACGGTGTCCTGATTGCGCATGACCTGCTGAAAGAAGGCGC | amplification of *ugd* downstream locus from genome |
| ugd-down-R | CCGATGACTCGAAGAGCCTCC |  |
| ugd-F | ATGAAAATTACTATTTCCGGTACAGG | knockout validation |
| ugd-R | GCCAGATAGGTGTTAGCGAA |  |
| wbgu-spacer-F | TAGTTCATACCAAGGTGAAACACC | *wbgU* spacer for gene deletion and replacement |
| wbgu-spacer-R | AAACGGTGTTTCACCTTGGTATGA |  |
| wbgu-up-F | CTGTTCAGTACAGCCATGCATCTCGTTACC | amplification of *wbgU* upstream locus from genome |
| wbgu-up-R | AGCGCCATATCCGGGCGACCGTTGTCGATTCCGACAACATCATGCCC |  |
| wbgu-down-F | ATGTTGTCGGAATCGACAACGGTCGCCCGGATATGGCGCT | amplification of *wbgU* downstream locus from genome |
| wbgu-down-R | GGTTTCAGGCGGGCGAGTATAAAGGC |  |
| wbgu-F | ATGAAGTTTTTGGTCACTGGTG | knockout validation |
| wbgu-R | GCTCTCAACGGTCCAGTCG |  |
| ugd-spacer-F/  wbgu-spacer-F | As above | successful cloning of the spacer |
| M13-R | CACACAGGAAACAGCTATGA |  |
| apr-F | AGCAGATCATCTCTGATCCA | Successful transformation of pSGKP |
| apr-R | TTCTGAAGGCTCTTCTCCTT |  |

Supplementary Table S6. SNPs between the genome sequence of the host strain and its phage-resistant mutants

| **strain** | **Contig no.** | **Positon** | **Type** | **Reference** | **Alteration** | **Region** | **Effect** | **Gene** | **Product** |  |
| --- | --- | --- | --- | --- | --- | --- | --- | --- | --- | --- |
|  |  |  |  |  |  |  |  |  |  |  |
| B1E1 | 113 | 240 | snp | C | T | CDS | missense mutation |  | mobile element |  |
|  | 10 | 165071 | complex | GT | CC | non-coding regions |  |  |  |  |
|  | 12 | 67368 | del | CCGCGG | C | non-coding regions |  |  |  |  |
|  | 12 | 67386 | complex | GGGGG | CGGTC | non-coding regions |  |  |  |  |
|  | 12 | 77232 | snp | C | T | CDS | missense mutation |  | hypothetical protein |  |
|  | 9 | 76705 | snp | C | A | CDS | missense mutation | *mdoD* | glucan synthesis-associated protein |  |
|  | 59 | 13703 | ins | C | CCTGCTA | CDS | frameshift mutation |  | hypothetical protein |  |
| B1E2 | 103 | 252 | snp | G | A | CDS | synonymous mutation |  | transposaseInsH |  |
|  | 113 | 240 | snp | C | T | CDS | missense mutation |  | mobile element |  |
|  | 12 | 67374 | complex | GGG | CGC | non-coding regions |  |  |  |  |
|  | 12 | 67390 | mnp | GC | CG | non-coding regions |  |  |  |  |
|  | 12 | 77232 | snp | C | A | CDS | missense mutation |  | hypothetical protein |  |
|  | 12 | 77240 | snp | T | A | CDS | missense mutation |  | hypothetical protein |  |
|  | 9 | 76705 | snp | C | A | CDS | missense mutation | *mdoD* | glucan synthesis-associated protein |  |
| B1E3 | 113 | 240 | snp | C | T | CDS | missense mutation |  | mobile element |  |
|  | 12 | 67374 | complex | GGGG | CGC | non-coding regions |  |  |  |  |
|  | 12 | 67386 | complex | GGGG | TGGT | non-coding regions |  |  |  |  |
|  | 12 | 77248 | snp | C | G | CDS | synonymous mutation |  | hypothetical protein |  |
|  | 6 | 37 | ins | C | CT | non-coding regions |  |  |  |  |
|  | 9 | 76705 | snp | C | A | CDS | missense mutation | *mdoD* | glucan synthesis-associated protein |  |
|  | 65 | 6732 | snp | C | T | CDS | synonymous mutation |  | transposaseInsH |  |
| B1G1 | 113 | 240 | snp | C | T | CDS | missense mutation |  | mobile element |  |
|  | 10 | 165328 | snp | A | G | non-coding regions |  |  |  |  |
|  | 12 | 67389 | complex | GGCC | CCG | non-coding regions |  |  |  |  |
|  | 19 | 98073 | snp | C | T | CDS | missense mutation | *btuB* | vitamin B12 transport protein |  |
|  | 65 | 6732 | snp | C | T | CDS | synonymous mutation |  | transfer protein TraK |  |
| B1G2 | 113 | 240 | snp | C | T | CDS | missense mutation |  | mobile element |  |
|  | 10 | 165328 | snp | A | G | non-coding regions |  |  |  |  |
|  | 12 | 67389 | complex | GGCC | CCG | non-coding regions |  |  |  |  |
|  | 19 | 98073 | snp | C | T | CDS | missense mutation | *btuB* | vitamin B12 transport protein |  |
| PR49 | 2 | 394201 | snp | C | A | non-coding regions |  |  |  |  |
|  | 15 | 2572 | snp | G | A | CDS | missense mutation | *btuB* | vitamin B12 transport protein |  |
|  | 31 | 439 | complex | TGTT | GTTA | non-coding regions |  |  |  |  |
|  | 31 | 474 | snp | G | A | non-coding regions |  |  |  |  |
| 140494A1 | 17 | 11253 | snp | G | T | CDS | missense mutation | *wcaJ* | glycosyltransferase |  |
| 140494A2 | 4 | 88013 | snp | A | G | non-coding regions |  |  |  |  |
|  | 17 | 11483 | snp | G | T | CDS | missense mutation | *wcaJ* | glycosyltransferase |  |
| 140494B2 | 86 | 96 | snp | C | T | non-coding regions |  |  |  |  |
| 140494C1 | 18 | 98292 | snp | G | T | CDS | missense mutation | *btuB* | vitamin B12 transport protein |  |
| 140494C2 | 18 | 98092 | snp | G | A | CDS | missense mutation | *btuB* | vitamin B12 transport protein |  |
|  | 86 | 96 | snp | C | T | non-coding regions |  |  |  |  |
| 140443A1 | 20 | 252 | snp | G | A | non-coding regions | synonymous mutation |  | IS5 family transposase IS*Kpn_26_* |  |
| 140443A2 | 20 | 252 | snp | G | A | non-coding regions | synonymous mutation |  | IS5 family transposase IS*Kpn_26_* |  |
| 140443C1 | 20 | 98049 | snp | C | T | CDS | missense mutation | *btuB* | vitamin B12 transport protein |  |
| Note：Coding sequence (CDS), single nucleotide polymorphism (snp), insertion (ins), deletion (del),Complex is defined as multiple mutations within ten nucleotides. | | | | | | | | | |  |

Supplementary Table S7. Pairwise nucleotide sequence identity (%) matrix of the *btuB* gene from cross-genus strains.

| Strain / Species | 135077 | 142486 | 170224 | 25922 | 38_AN |
| --- | --- | --- | --- | --- | --- |
| 135077 / *Klebsiella pneumoniae* | 100.00 | 78.79 | 63.21 | 62.61 | 61.79 |
| 142486 / *Kluyvera tianfuensis* | 78.79 | 100.00 | 63.70 | 61.84 | 62.88 |
| 170224 / *Enterobacter ludwigii* | 63.21 | 63.70 | 100.00 | 72.80 | 72.64 |
| 25922 / *Escherichia coli* | 62.61 | 61.84 | 72.80 | 100.00 | 79.67 |
| 38_AN / *Salmonella Typhimurium* | 61.79 | 62.88 | 72.64 | 79.67 | 100.00 |

Supplementary Table S8. Results of global structural alignment of BtuB homologs.

| Target structure vs. reference (135077) | Residues aligned | RMSD (Å) |
| --- | --- | --- |
| ATCC25922 | 614 vs 612 | 0.638 |
| 142486 | 618 vs 612 | 0.309 |
| 170224 | 618 vs 612 | 0.648 |
| 38_AN | 614 vs 612 | 0.538 |

Supplementary Table S9. Pairwise nucleotide sequence identity (%) matrix of *wcaJ*, *wbaZ*, *ugd*, and *wbgU* genes from ST11-KL64 CRKP in our study.

| Strain | 135077 | 140531 | 140443 | 140494 | 140077 | 135080 | 135055 | 135044 | 135025 | 135018 | 015134 |
| --- | --- | --- | --- | --- | --- | --- | --- | --- | --- | --- | --- |
| 135077 | 100 | 100 | 100 | 100 | 100 | 100 | 100 | 100 | 100 | 100 | 100 |
| 140531 | 100 | 100 | 100 | 100 | 100 | 100 | 100 | 100 | 100 | 100 | 100 |
| 140443 | 100 | 100 | 100 | 100 | 100 | 100 | 100 | 100 | 100 | 100 | 100 |
| 140494 | 100 | 100 | 100 | 100 | 100 | 100 | 100 | 100 | 100 | 100 | 100 |
| 140077 | 100 | 100 | 100 | 100 | 100 | 100 | 100 | 100 | 100 | 100 | 100 |
| 135080 | 100 | 100 | 100 | 100 | 100 | 100 | 100 | 100 | 100 | 100 | 100 |
| 135055 | 100 | 100 | 100 | 100 | 100 | 100 | 100 | 100 | 100 | 100 | 100 |
| 135044 | 100 | 100 | 100 | 100 | 100 | 100 | 100 | 100 | 100 | 100 | 100 |
| 135025 | 100 | 100 | 100 | 100 | 100 | 100 | 100 | 100 | 100 | 100 | 100 |
| 135018 | 100 | 100 | 100 | 100 | 100 | 100 | 100 | 100 | 100 | 100 | 100 |
| 015134 | 100 | 100 | 100 | 100 | 100 | 100 | 100 | 100 | 100 | 100 | 100 |

Supplementary Table S10. The accession numbers of other phage-resistant mutants are mentioned in this study.

| **Strain** | **Source** | **Accession no.** |
| --- | --- | --- |
| 140443A1 | 140443+P04 | JBUYSK000000000 |
| 140443A2 | 140443+P04 | JBUYSJ000000000 |
| 140443B1 | 140443+P04+P40 | JBUYSI000000000 |
| 140443B2 | 140443+P04+P40 | JBUYSH000000000 |
| 140443C1 | 140443+P04+P40+P49 | JBUYSG000000000 |
| 140443C2 | 140443+P04+P40+P49 | JBUYSF000000000 |
| 140494A1 | 140494+P04 | JBUYSE000000000 |
| 140494A2 | 140494+P04 | JBUYSD000000000 |
| 140494B1 | 140494+P04+P40 | JBUYSC000000000 |
| 140494B2 | 140494+P04+P40 | JBUYSB000000000 |
| 140494C1 | 140494+P04+P40+P49 | JBUYSA000000000 |
| 140494C2 | 140494+P04+P40+P49 | JBUYRZ000000000 |
| PR49 | 170224+P49 | JBUYSL000000000 |

**Supplementary Figure S1. Biological characterization of P40 and P49.**

(A) P40 produced the highest amount of progeny phages when the multiplicity of infection (MOI, the ratio of phage titer to the number of host bacteria) was 1. (B) P49 had an optimal MOI of 0.1. (C) The adsorption rates. Within 15 min, >90% of P49 particles were rapidly adsorbed to strain B1E2 and more than 90% of P40 was rapidly adsorbed to strain PR1 within 15 min. (D) One-step growth curve. The latent period of P40 was approximately 50 min according to the one-step growth curve with a burst size of 112 ± 8 progeny phages per infected bacterial cell. P49 has a latent period of approximately 30 min, and a burst size of 157 ± 48 progeny phages per infected bacterial cell. (E) The stability of phages at various temperatures. P40 and P49 were stable in solution at 0 to 50 °C. (F) The stability of phages under different pH values. Values represent the mean ± standard deviations and all experiments were biologically in triplicate.


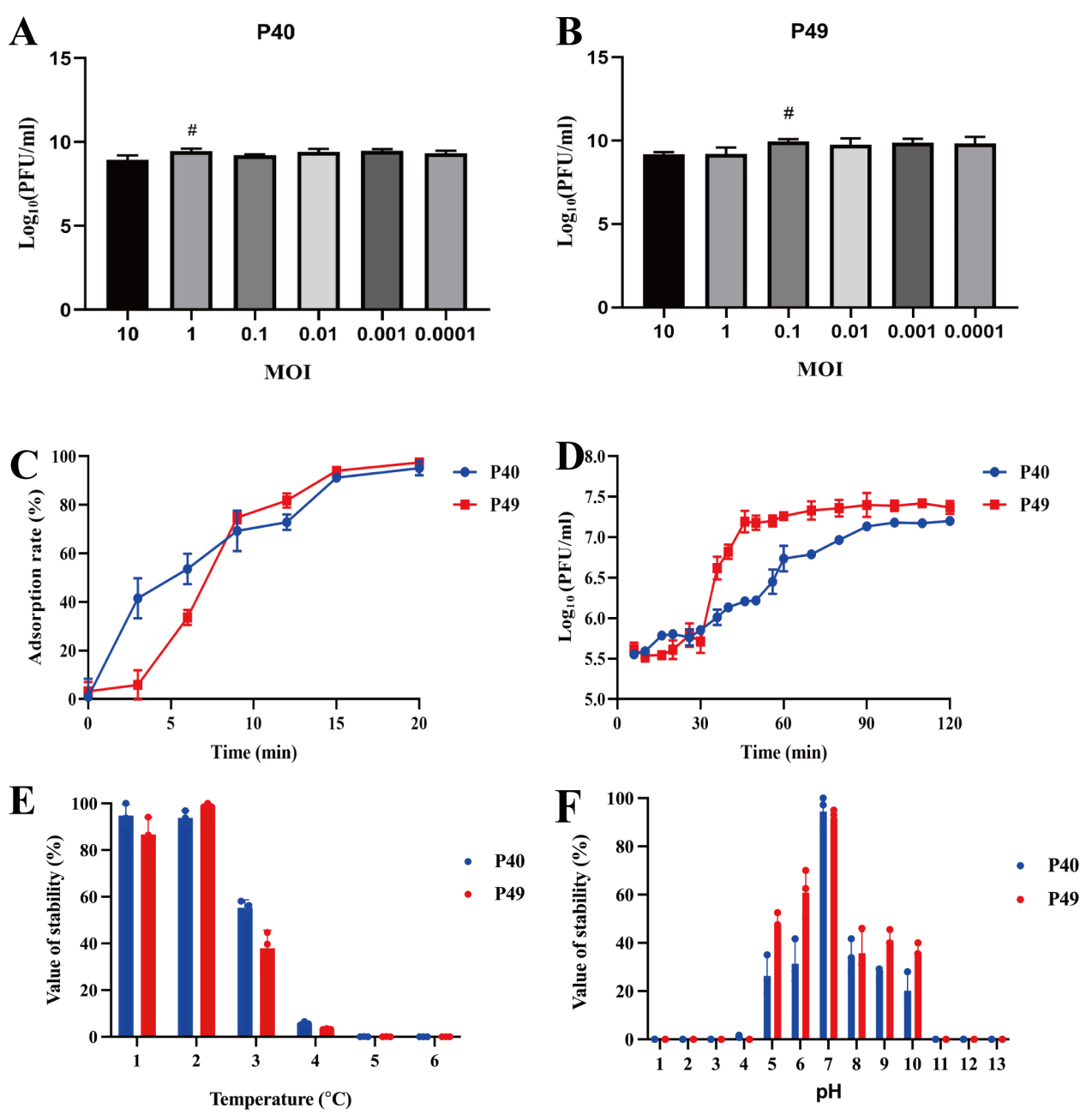


**Supplementary Figure S2. The phylogenetic tree of phage P40.**

The phylogenetic tree of phages belonging to the genus *Slopekvirus* within the family *Strabovirida*e based on a terminase large subunit according to ICTV.

**
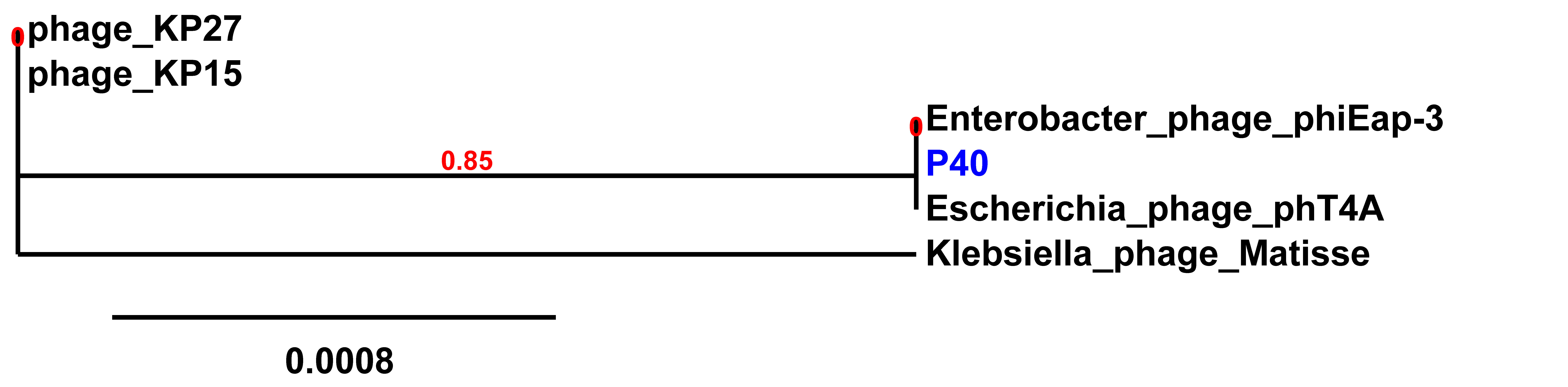
**

**Supplementary Figure S3. The phylogenetic tree of phage P49.**

The phylogenetic tree of P49 belonging to the genus *Epseptimavirus* within the family *Demerecviridae* based on a terminase large subunit according to ICTV. Black font represents *Salmonella* phages and green font represents *Escheichia* phages. Blue font represents different genera of the *Demerecviridae* family. The abovementioned genera are included in the subfamily *Markadamsvirinae*, belonging to the family *Demerecviridae*.


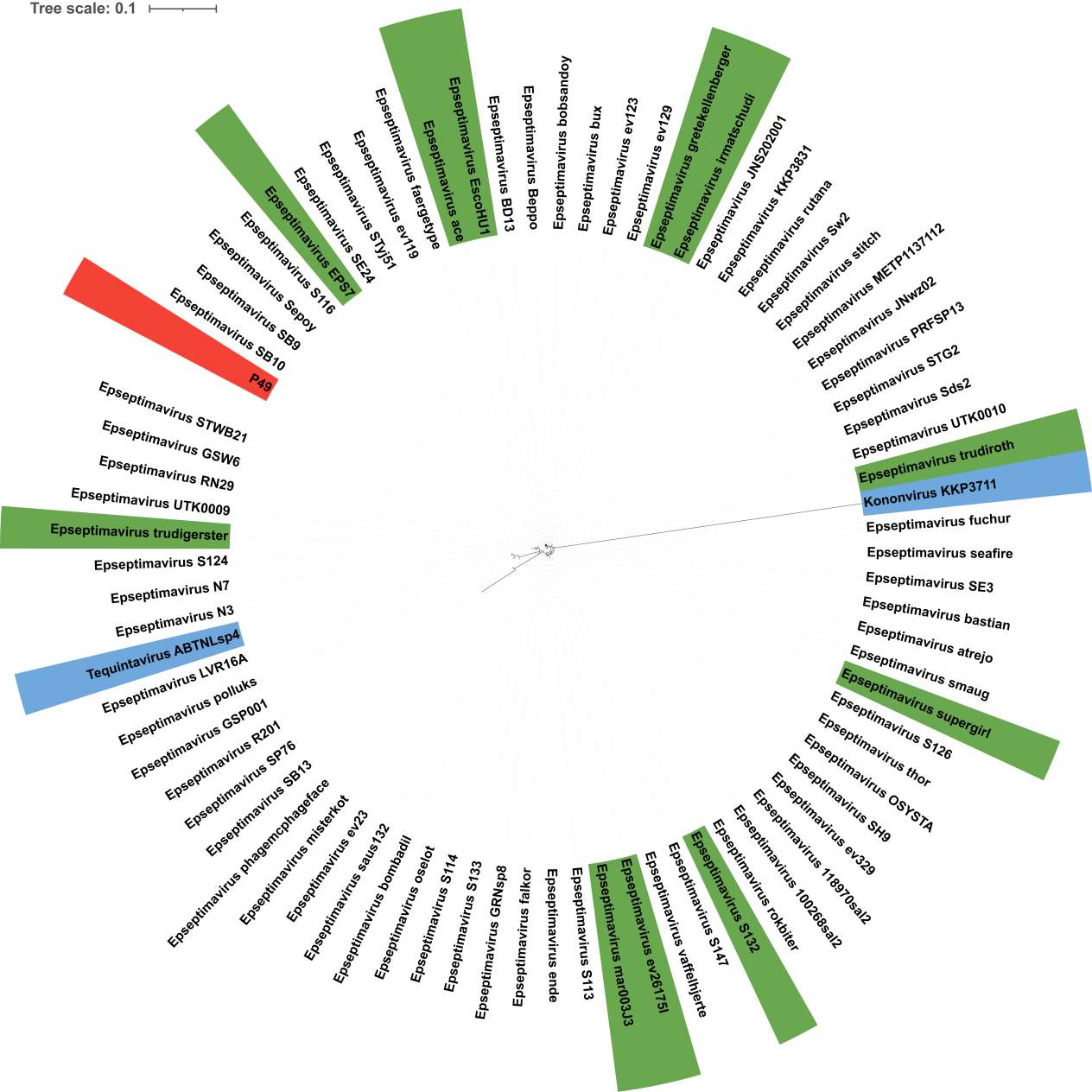


**Supplementary Figure S4. Phage P40 susceptibility tests targeting B1E2 and B1E3 complemented with *ugd* and *wbgU* alone.**

The plaque assay method confirmed that P40 could not lyse B1E2 and B1E3 complemented with *ugd* and *wbgU* alone. Phage suspensions (5 μl, approximately 10^9^ PFU/ml) was dropped onto the plates and incubated at 37 °C.


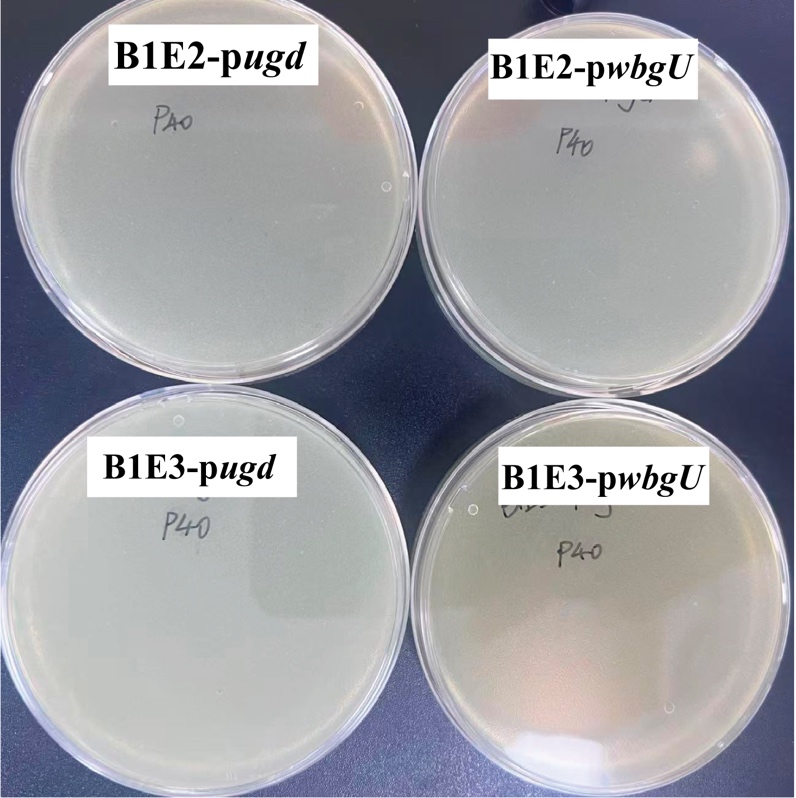


**Supplementary Figure S5. Phage P40 susceptibility tests targeting P04/P40-resistant mutants after gene complementation.**

The P04/P40-resistant mutants after gene complementation (*gnd*, *rmlA,* *rmlB*, *rmlC*, *rmlD*, *manB*, *manC*, and *acyT*) did not restore sensitivity to P40. Phage suspensions (5 μl, approximately 10^9^ PFU/ml) was dropped onto the plates and incubated at 37 °C.


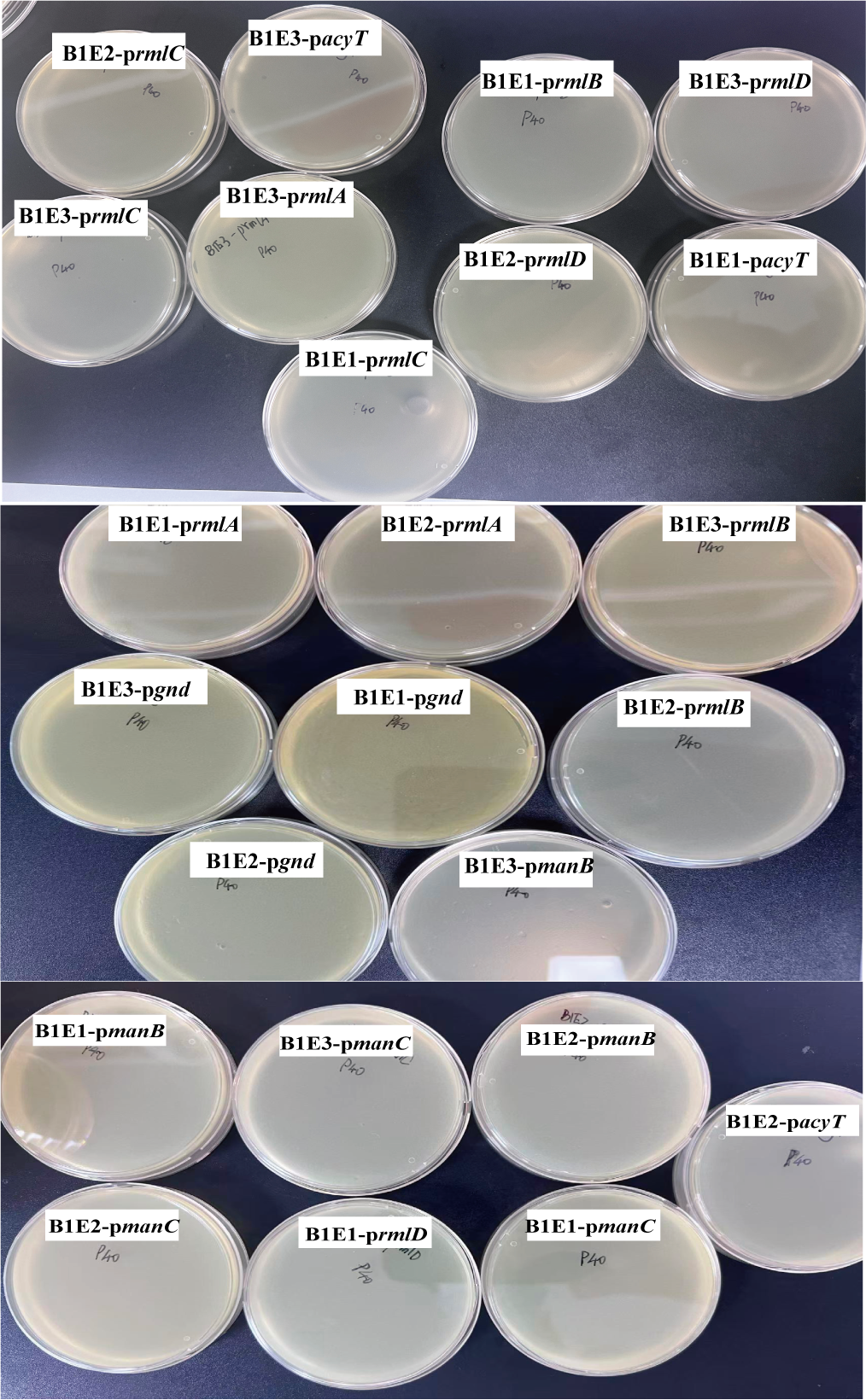


**Supplementary Figure S6. Phage P40 susceptibility tests targeting P04/P40-resistant mutants after gene knockout and complementation.**

(A) P40 and P49 could lyse PR1. (B) P40 could not lyse PR1 strain after *wbgU* gene knockout. (C) P40 could not lyse PR1 strain after *ugd* gene knockout. (D, E) The knockout strain after the corresponding gene complementation restored the sensitivity to P40.

**
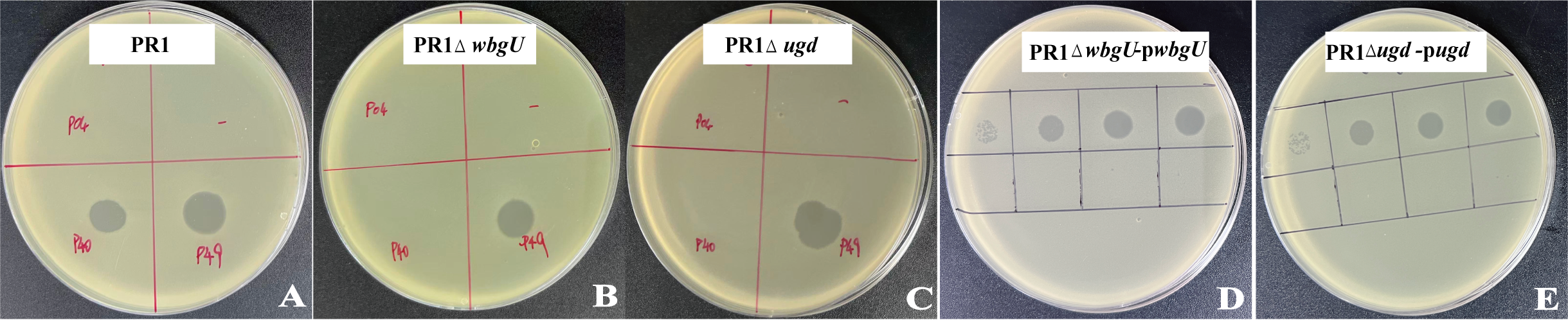
**

**Supplementary Figure S7. Phage adsorption rate.**

1. Adsorption rate of phage P40. P40 could not adsorb onto the P04/P40-resistant mutants. About 90% of the P40 were adsorbed to P04/P40-resistant mutants after genes complementation at 20 min. (B) Adsorption rate of phage P49. P49 could not adsorb onto the P04/P40/P49-resistant mutants. About 90% of the P49 were adsorbed to P04/P40/P49-resistant mutants after gene complementation at 20 min.


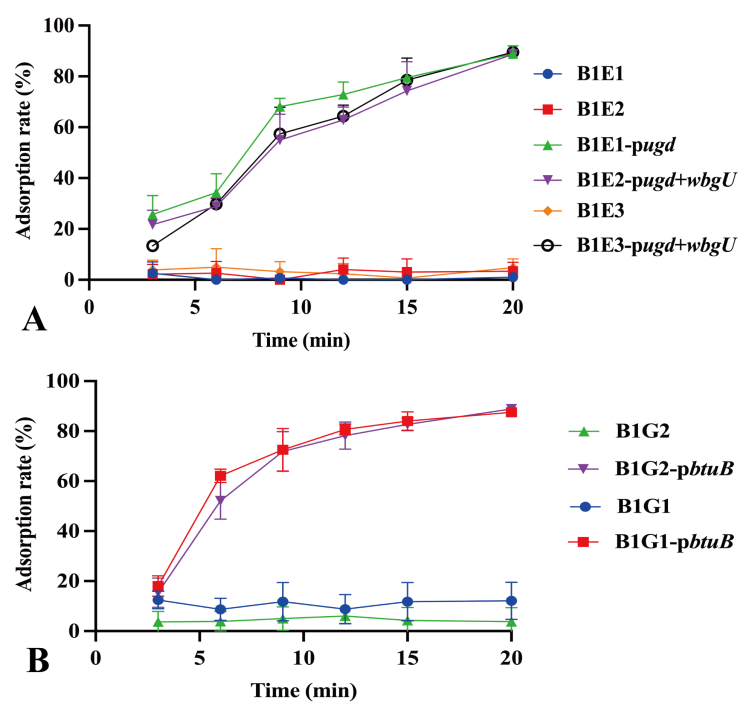


**Supplementary Figure S8. The homologous recombination of two copies of IS*Kpn26* resulted in the loss of the 22-kb fragment in B1E1.**

Firstly, the direct copy of IS*Kpn26* was inserted between *wzb* and *wzc* genes. Then, homologous recombination between two IS*Kpn26* resulted in the loss of a large fragment. The *wzb*-IS*Kpn26*-*wbgU* structure existed in B1E1.


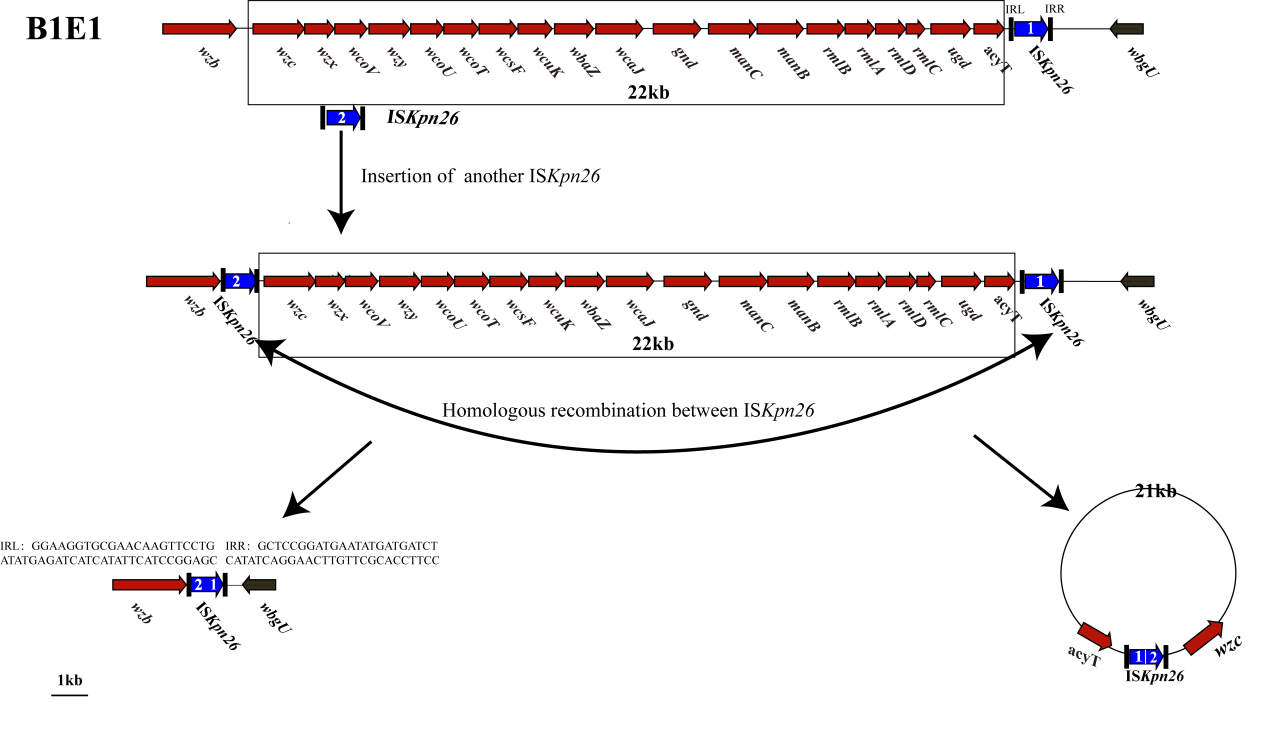


**Supplementary Figure S9. The homologous recombination of two copies of IS*903B* resulted in the loss of the 26-kb fragment in B1E2.**

Firstly, the direct copy of IS*903B* was inserted into the *wzx* gene. Then, homologous recombination between two IS*903B* resulted in the loss of a large fragment. The *wzx*Δ-IS*903B*-*wbbM* structure existed in B1E2.


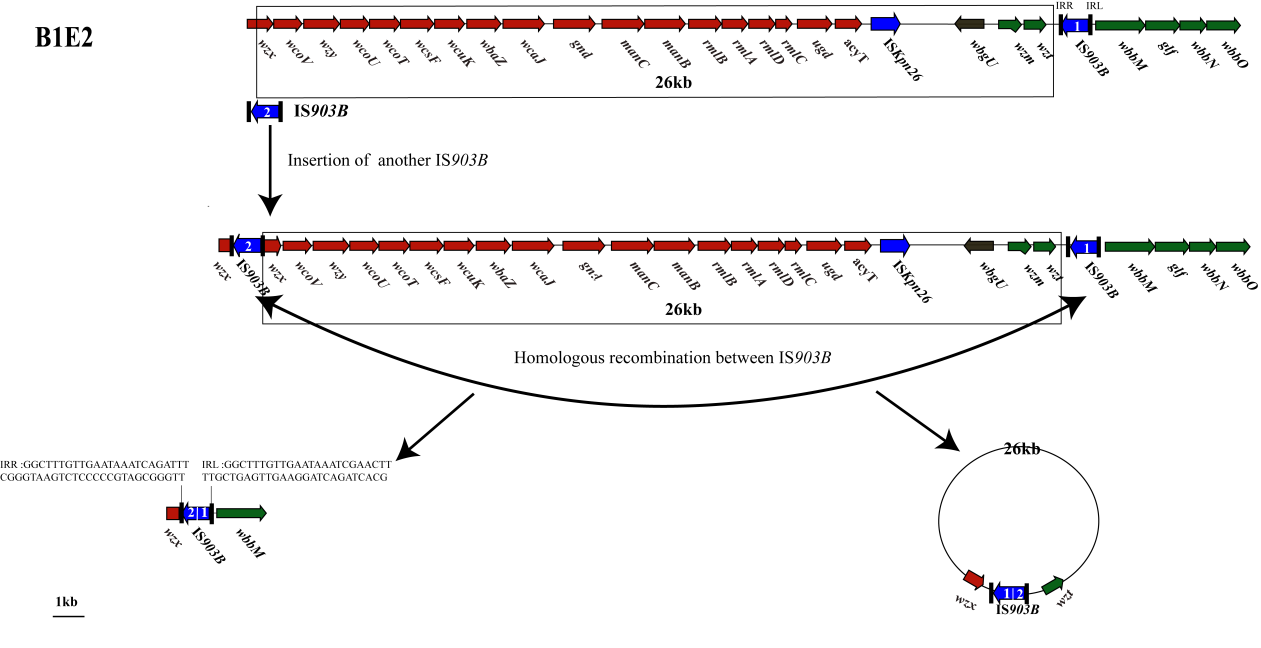


**Supplementary Figure S10. The homologous recombination of two copies of IS*903B* resulted in the loss of the 18-kb fragment in B1E3.**

Firstly, the direct copy of IS*903B* was inserted into a *wcaJ* gene. Then, homologous recombination between two IS*903B* resulted in the loss of large fragment. The *wcaJ*Δ-IS*903B*-*wbbM* structure existed in B1E3.


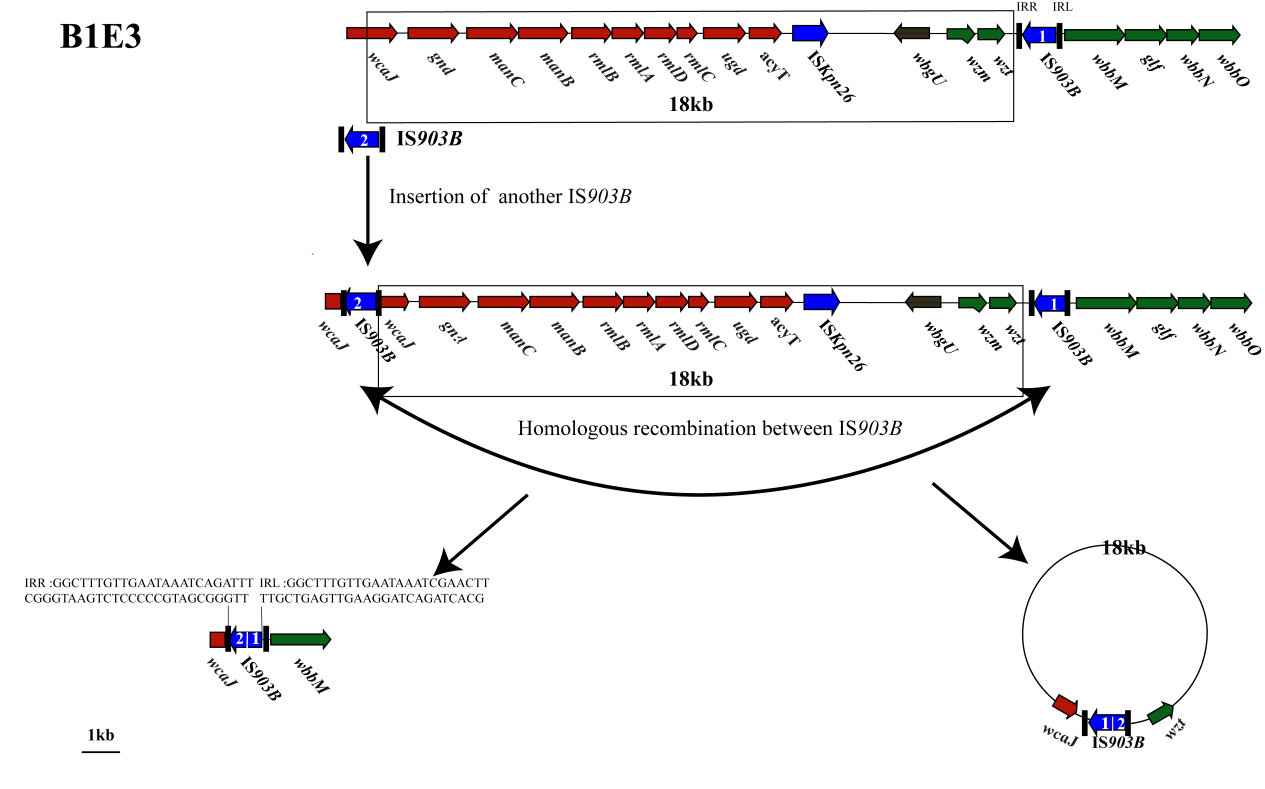


**Supplementary Figure S11. The homologous recombination of two copies of IS*903B* resulted in the loss of the 52-kb fragment in B1G1 and B1G2.**

Firstly, the direct copy of IS*903B* was inserted into between *mdtA* (encoding multidrug efflux RND transporter periplasmic adaptor subunit) and *gltp* (encoding a dicarboxylate/amino acid-cation symporter) genes. Then, homologous recombination between two IS*903B* resulted in the loss of large fragment. The *mdtA*-IS*903B*-*wbbM* structure existed in B1G1 and B1G2.


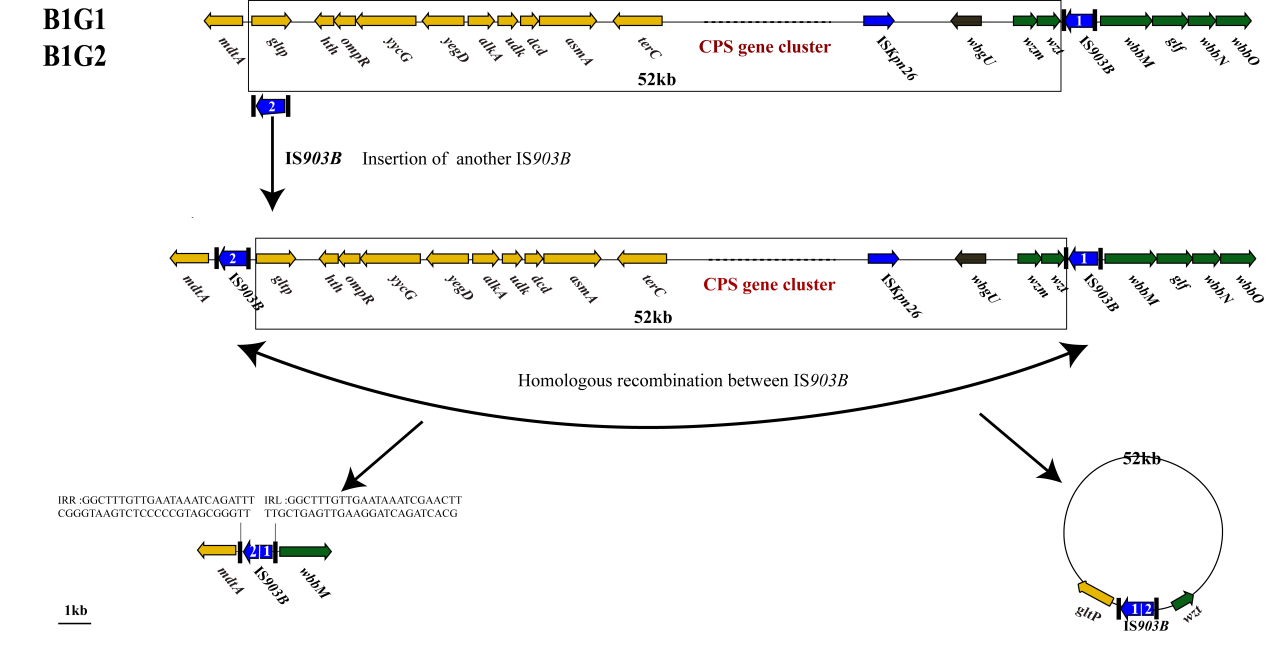


**Supplementary Figure S12. The lost genetic regions in P04/P40-resistant mutants and P04/P40/P49-resistant mutants were identified by coculturing the other ST11-KL64 CRKP with the phage cocktail.**

(A) The lost genetic regions in phage cocktail resistance mutants were identified by coculturing 140494 with the phage cocktail. The lost genetic regions in P04/P40/P49-resistant mutants are shown in the red box. The lost genetic regions in P04/P40-resistant mutants are shown in the black box. (B) The lost genetic regions in phage cocktail resistance mutants were identified by coculturing 140443 with the phage cocktail. The missing genetic regions are highlighted with boxes.


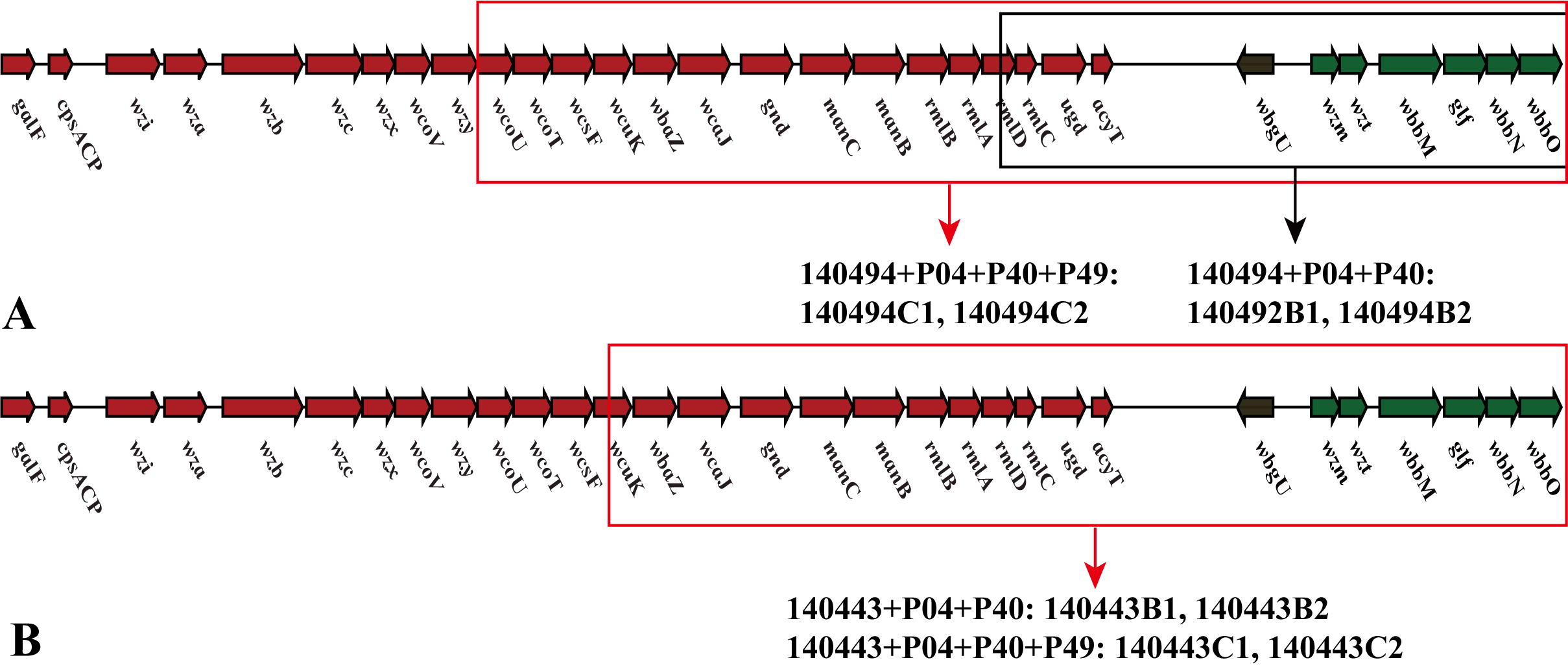

Supplement: Supplementary material clean.docx [file TEMI_A_2648890_SM4239.docx]
